# Loss of BATF3 impairs adipose-liver homeostasis and accelerates the transition from steatosis to fibrosis in high-fat diet-fed mice

**DOI:** 10.7150/ijbs.128084

**Published:** 2026-04-16

**Authors:** Haibo Dong, Liuyi Hao, Wei Guo, Xinguo Sun, Zhanxiang Zhou

**Affiliations:** 1 Center for Translational Biomedical Research, the University of North Carolina at Greensboro, North Carolina Research Campus, Kannapolis, NC, USA.; 2 Department of Nutrition, the University of North Carolina at Greensboro, Greensboro, NC, USA.

**Keywords:** BATF3, hepatic fibrosis, insulin resistance, metabolic inflammation, MASLD, ANGPTL8

## Abstract

BATF3 is a transcription factor critical for dendritic cell differentiation and immune regulation. Although recent studies suggest that BATF3 is involved in metabolic disorders, the mechanism by which BATF3 deficiency contributes to the development of metabolic dysfunction-associated fatty liver disease (MASLD) remains unclear. Here, we examined the impact of BATF3 deficiency in mice fed a high-fat diet (HFD). We discovered that BATF3 is essential for maintaining metabolic homeostasis in adipose tissue and liver. Batf3⁻/⁻ mice developed aggravated hepatic steatosis, inflammation, and fibrosis, accompanied by enhanced adipose lipolysis, increased hepatic fatty acid uptake, and impaired insulin-AKT signaling. Our investigations into gene expression affected by BATF3 deficiency showed that angiopoietin-like protein 8 (ANGPTL8), a hepatokine abundantly expressed in the liver and adipose tissue, was specifically downregulated, identifying ANGPTL8 as a key mediator of BATF3-regulated hepatic and adipose tissue homeostasis. Importantly, we found that in addition to directly inhibiting inflammation-induced hepatic stellate cell activation, ANGPTL8 also has tissue-specific effects on lipid metabolism, alleviating hepatic lipid deposition and suppressing adipose tissue lipolysis. Collectively, our findings provide mechanistic insights into how BATF3 regulates hepatic and adipose homeostasis, contributing to fibrosis development, and highlight the BATF3-ANGPTL8 axis as a potential therapeutic target in fatty liver disease.

## 1. Introduction

Metabolic dysfunction-associated steatotic liver disease (MASLD, formerly known as NAFLD) has become the most prevalent chronic liver condition worldwide 1-3, spanning a spectrum from simple steatosis to steatohepatitis, progressive fibrosis, cirrhosis, and hepatocellular carcinoma 4. MASLD is closely associated with central obesity, systemic insulin resistance, and cardiometabolic risk, which underscores the clinical and societal burden of the disease. Although early studies emphasized a stepwise evolution from lipid accumulation to inflammatory injury, current concepts support a “multiple-hit” paradigm in which dysregulated lipid handling, innate and adaptive immune activation, and organ-to-organ communication act in parallel to drive disease progression 5. A persistent challenge is to delineate which immune-metabolic cues govern the transition from benign steatosis to fibrogenic steatohepatitis and why such progression only occurs in a subset of individuals 6. Clarifying these determinants is essential for mechanism-based therapies that can interrupt the feed-forward loops among lipid flux, inflammation, and tissue injury.

Adipose-liver crosstalk is a key conduit through which systemic metabolic stress is translated into hepatic injury 7. Inflamed adipose tissue exhibits macrophage aggregation around dying adipocytes (crown-like structures), neutrophil recruitment, and production of pro-inflammatory cytokines that amplify local lipolysis and release of free fatty acids (FFAs) into the circulation 8. Hepatic uptake of these FFAs promotes ectopic triglyceride storage, oxidative stress, and innate immune activation, establishing a vicious cycle that sustains steatohepatitis and sensitizes to fibrogenesis. Although this mechanism is recognized, the upstream regulators that maintain tissue homeostasis under physiological conditions and restrain inflammatory amplification during dietary stress remain poorly defined. In addition, hepatokines have emerged as critical endocrine mediators that orchestrate communication between the liver and peripheral metabolic tissues, including adipose tissue. By modulating lipid handling, glucose metabolism, and inflammatory responses, hepatokines provide a systemic signal of hepatic status to distal organs and, in turn, integrate peripheral cues back to the liver. However, accumulating evidence indicates that the regulation of hepatokine expression is highly context- and tissue-dependent, varying with nutritional state, metabolic stress, and inflammatory milieu. As a result, their precise functional roles in coupling adipose inflammation to hepatic injury remain incompletely understood, and some studies show that the results across different experimental models are contradictory 9-12. These uncertainties underscore the need to clarify how defects in immune-metabolic balance reshape adipose-liver communication and accelerate the transition from steatosis to steatohepatitis and fibrosis.

Basic leucine zipper ATF-like 3 (BATF3) is essential for the development of conventional type 1 dendritic cells (cDC1s), a lineage crucial for antigen presentation and regulation of tissue immune tone 13. Work across immunity, infection, and tumor biology has established BATF3-dependent cDC1s as pivotal sentinels that shape local inflammatory milieus 14, 15. Emerging evidence extend these insights to metabolism, suggesting that perturbations in BATF3-dependent immune circuits can influence systemic metabolic homeostasis, such as adipose function, intestinal epithelial integrity, and susceptibility to metabolic syndrome 16, 17. Notably, dendritic cell-centered studies in liver disease suggest that specific DC subsets can modulate steatosis and inflammation 18. However, the impact of BATF3-dependent programs on the adipose-liver axis, insulin signaling, and fibrogenic progression under dietary stress remains undefined.

Here, we aimed to investigate the role of BATF3 in the development and progression of metabolic dysfunction-associated steatotic liver disease (MASLD). Specifically, we examined whether BATF3 deficiency influences adipose tissue and hepatic inflammation, lipid trafficking, insulin signaling, and fibrogenesis, as well as hepatokine expression under dietary stress. Particularly, angiopoietin-like protein 8 (ANGPTL8), a hepatokine expressed in both liver and adipose tissue, emerged in our study as a key mediator linking BATF3 deficiency to altered lipid metabolism and fibrogenic responses. Collectively, our findings demonstrate that loss of BATF3 aggravates adipose-liver crosstalk by enhancing metabolic inflammation and reducing ANGPTL8 expression, thereby linking immune imbalance to the progression of diet-induced steatohepatitis and fibrosis.

## 2. Materials and Methods

### Animals and high-fat diet feeding

C57BL/6J mice (WT) and *Batf3* knockout (*Batf3*^-/-^, JAX stock number: 013755) mice were purchased from the Jackson Laboratory (Bar Harbor, ME). These *Batf3^-/-^* mutant mice lack exons 1-2 of the basic leucine zipper transcription factor, ATF-like 3 (Batf3), thereby abolishing gene function 19. All mice were housed under specific pathogen-free conditions with controlled temperature and a 12-hour light/dark cycle. For the collection of tissue samples, mice were anesthetized with inhalational isoflurane. All animal studies were conducted by following the protocol approved by the North Carolina Research Campus Institutional Animal Care and Use Committee. Metabolic dysfunction-associated fatty liver disease (MASLD) model was produced by using a rodent High-fat diet (HFD) with 45% kcal fat (Research Diet, D12451) and standard normal chow diet (Chow) as control. Eight-week-old male WT and *Batf3*^-/-^ mice were fed the control or HFD for 16 weeks.

### Tissue collection and histopathology

At sacrifice, mice were anesthetized with isoflurane, and blood, liver, and gonadal white adipose tissue (gWAT) were collected. Organs were weighed, snap-frozen in liquid nitrogen, or fixed in 4% paraformaldehyde (PFA) for histological analysis. Paraffin-embedded liver and gWAT sections (5 μm) were stained with hematoxylin and eosin (H&E) to assess lipid droplet accumulation and crown-like structures (CLSs). Periodic Acid Schiff (PAS) staining was performed to visualize glycogen deposition.

### Cell culture and treatment

Human hepatoma cells (HepG2), murine adipocytes (3T3-L1), and human hepatic stellate cells (LX-2) were obtained from ATCC and maintained under recommended conditions. Differentiation of 3T3-L1 cells into adipocytes was induced using a standard cocktail of insulin, dexamethasone, and IBMX. Cells were plated in 6-well plates and incubated with a wide range of concentrations of rhANGPTL8 (ng/well) with free fatty acids (FFA- palmitate, 200 μM) or human TNFα (rhTNFα, 50ng/well). Intracellular TG accumulation was measured by BODIPY staining or TG assay kits. Glycerol release in adipocyte supernatants was measured as an indicator of lipolysis. LX-2 stellate cell activation was assessed by Collagen I expression. To silence TNF-R1 in HepG2, a siRNA targeting *Tnfrsf1a* (sc-29507, Santa Cruz Biotechnology) was used.

### RNA isolation and real-time PCR

Total RNA from liver, adipose tissue, HepG2, and 3T3-L1 was extracted with TRIzol reagent (Invitrogen, Oregon, USA). cDNA was synthesized using a reverse transcription kit (Applied Biosystems). Quantitative PCR (qPCR) was performed with SYBR Green Master Mix (Applied Biosystems) on a QuantStudio 5 Flex Real-Time PCR System. Expression of target genes related to inflammation, fibrosis, lipid metabolism, and hepatokines was normalized to *RPS17* (for mouse) or *18s* (for human). Relative expression was calculated using the 2^-ΔΔCt method. Primers used for real-time PCR are depicted in Supplementary Table 1.

### Flow cytometry analyses

Single-cell suspensions from liver and gWAT were prepared by mechanical dissociation and enzymatic digestion with collagenase P (200 μg/mL, Sigma, #11249002001), Dispase (800 μg/mL, Sigma, #D4693) and DNase I (100 μg/mL, Sigma, #10104159001) for 60 min at 37 °C. Cells were filtered through 70 μm strainers, washed, and resuspended in FACS buffer. After Fc receptor blocking (anti-CD16/CD32), cells were stained with fluorophore-conjugated antibodies: CD45, CD11b, Ly6C, and Ly6G (all from BioLegend) for 25 minutes of incubation in the dark on ice, and then 7-AAD (5 μl/per test) was added for another 5 min incubation at room temperature to exclude dead cells. Flow cytometry using a fluorescence-activated cell sorting (FACS) Melody analyzer (BD Life Sciences), and data were analyzed with Flowjo 10.1 software (BD Life Sciences). Monocytes were defined as CD45⁺CD11b⁺Ly6C⁺, and neutrophils as CD45⁺CD11b⁺Ly6G⁺ populations. All antibodies are listed in Supplementary Table 2.

### Immunohistochemistry (IHC), and immunofluorescence (IF)

Paraffin or cryosections were deparaffinized, rehydrated, and subjected to antigen retrieval. Endogenous peroxidase activity was quenched with 3% hydrogen peroxide, followed by blocking with 2.5% normal goat serum (for IHC) or 10% normal donkey serum (for IF). Sections were incubated with primary antibodies against myeloperoxidase (MPO; neutrophil marker), F4/80 (macrophage marker), CHOP, 4-HNE (oxidative stress marker), Collagen I (fibrosis marker), p-AKT (insulin resistance marker) and ANGPTL8 (hepatokine) overnight at 4 °C. After washing, slides were incubated with HRP-conjugated secondary antibodies for IHC or fluorescently labeled secondary antibodies for IF. Signals were visualized with DAB (IHC) or immunofluorescence microscopy (IF, Nikon/NIS-Elements). All antibodies are listed in Supplementary Table 2.

### Measurement of MCP1 by ELISA

MCP1 protein levels in mouse liver and gonadal white adipose tissue (gWAT) were quantified using a commercial Quantikine® sandwich ELISA kit (# MJE00B; R&D Systems) following the manufacturer's instructions. Tissue lysates were prepared using the lysis buffers recommended for this assay and clarified by centrifugation before analysis. All reagents and samples were brought to room temperature before use, and standards/controls/samples were assayed in duplicate. Briefly, 50 μL Assay Diluent RD1W was added to each pre-coated well, followed by 50 μL standard/control/sample and incubation for 2 h at room temperature. Wells were then washed four times with wash buffer. Next, 100 μL MCP1 conjugate was added and incubated for 2 h at room temperature, followed by repeat washing. 100 μL substrate solution (prepared by mixing Color Reagent A and B at equal volumes within 15 min of use) was added and incubated for 30 min at room temperature, protected from light. The reaction was stopped with 100 μL stop solution, and absorbance was read at 450 nm.

### Liver triglyceride (TG) assay

The TG levels in the liver and HepG2 cells were measured with colorimetric assay kits (Abcam, #ab65336). Briefly, 100mg tissues or 5 x 10^6 cells were harvested and lysed in 5% v/v NP-40, then samples were repeatedly heated at 98°C to solubilize triglycerides; a sample was taken for BCA protein quantification as a normalizer, and the indicated amount of sample was processed with lipase and reaction reagents. Colorimetric assay at 570 nm by using CYTATION 5 imaging reader (Biotek).

### Serum free fatty acid (FFA) measurement

Serum FFA levels were quantified using a commercial Free Fatty Acid Assay Kit (#ab65341, Abcam) according to the manufacturer's instructions. Briefly, serum samples were assayed after dilution in the supplied assay buffer to ensure readings fell within the standard curve range. For each well, 2 μL ACS reagent was added and incubated at 37°C for 30 min, followed by addition of 50 μL reaction mix and incubation at 37 °C for 30 min protected from light. Absorbance was measured at 570 nm, and FFA concentrations were calculated from the standard curve with blank subtraction and correction for dilution factors.

### Lipolysis measurement

The 3T3-L1 adipocytes (on day 8 post-differentiation) were plated in 6-well plates and incubated with a wide range of concentrations of rhANGPTL8 (10 ng/well, 50 ng/well, and 100 ng/well, R&D Systems, #10159-AN) in the presence or absence of rhTNFα (50 ng/well, R&D Systems, #10291-TA). Medium was collected at the end of 24 h for the measurement of glycerol release. Glycerol concentrations were determined by using a free glycerol determination kit according to the manufacturer's protocol (Sigma-Aldrich, #FG0100). Data was reported as a fold increase over levels of untreated cells.

### VLDL (very low-density lipoprotein) secretion rate assay

16 hours-fasted mice were injected with Triton WR1339 (500 mg/kg in saline), a lipoprotein lipase inhibitor that inhibits VLDL hydrolysis, via the Retro-orbital. VLDL secretion rate was calculated by subtracting triglyceride levels at 0 min from their counterpart levels at 90 min.

### Lipid droplets staining

Neutral lipid droplets in HepG2 cells or differentiated 3T3-L1 cells were stained using boron-dipyrromethene (BODIPY493/503). Briefly, the live cells were incubated with 2 μM BODIPY in PBS for 15 min at 37 ℃. After that, wash twice with PBS and then fixed in 4% formaldehyde for 15 min at room temperature.

### Oral glucose tolerance tests (OGTT)

After a 6-hour fast, mice underwent an oral glucose tolerance test (OGTT) with an oral glucose dose (2 g/kg). Blood samples were collected from the tail vein at 0, 15, 30, 60, and 120 minutes post-glucose administration. Blood glucose levels were measured using a blood glucose meter (Nova Biomedical).

### Statistical analysis

Data that met with normal distribution were analyzed using the independent-samples t-test or one-way analysis of variance followed by LSD's multiple comparison. Data that do not conform to a normal distribution were analyzed using Nonparametric tests followed by the Median test (K samples). Statistical analyses were performed using SPSS 21. Data were expressed as mean ± SD. In all tests, *P* < 0.05 was taken as significant.

## 3. Results

### BATF3 deficiency induces lipid accumulation and inflammation in the adipose tissue and liver under basal dietary conditions

**Figure 1:**
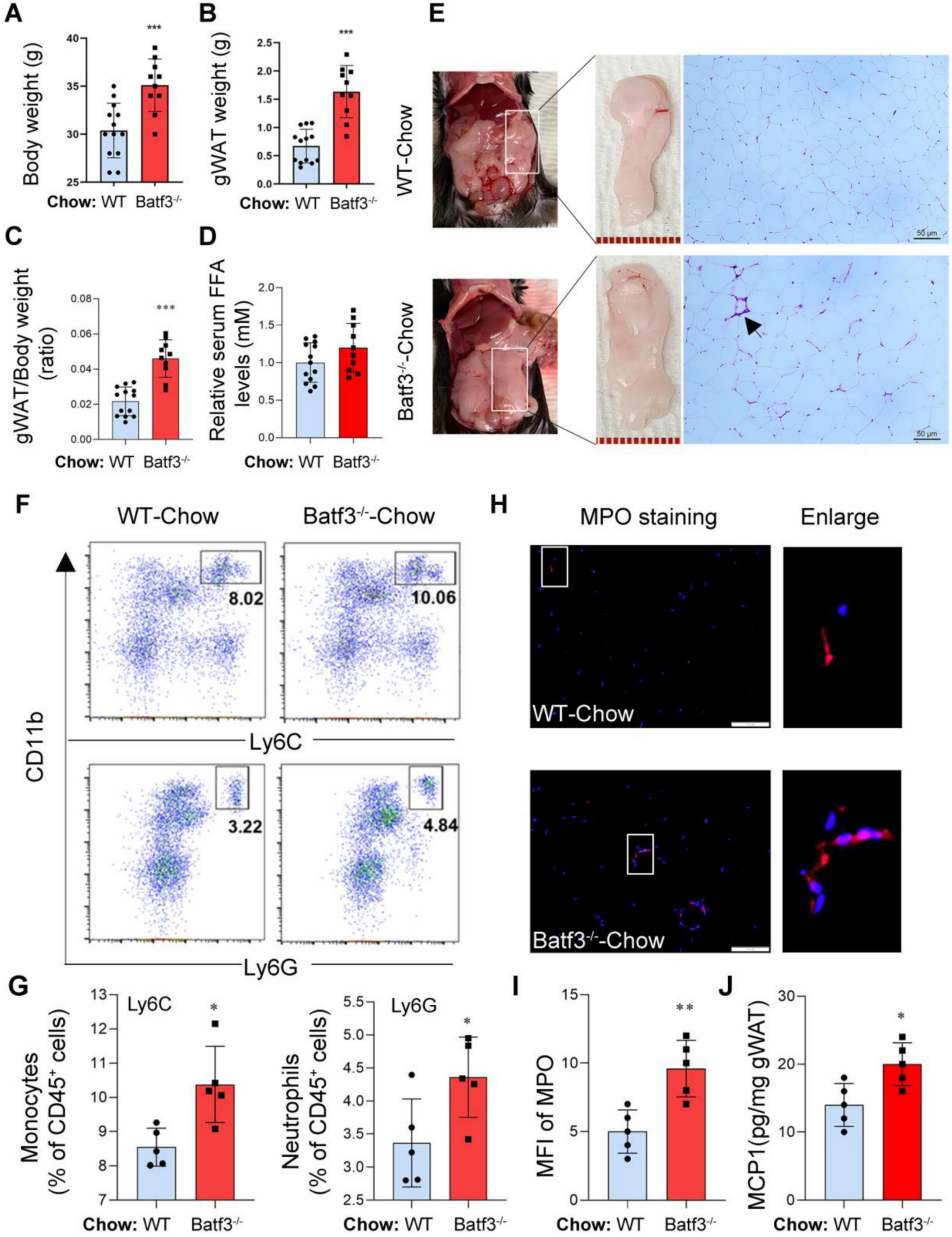
** BATF3 deficiency induces adipose tissue inflammation under normal chow diet conditions. (A-C)** Body weight, gonadal white adipose tissue (gWAT) mass, and gWAT/body weight ratio were significantly increased in Batf3⁻/⁻ mice compared with wild-type (WT) controls, indicating an obesity phenotype under basal conditions. **(D)** Serum free fatty acid (FFA) levels were measured to evaluate systemic lipid mobilization. No significant difference was observed between WT and Batf3⁻/⁻ mice under normal chow conditions. **(E)** H&E staining revealed increased crown-like structures (CLSs, black arrow) in gWAT of Batf3⁻/⁻ mice, consistent with macrophage infiltration. Scale bar, 50 μm. **(F-G)** Flow cytometry showed elevated CD11b⁺Ly6C⁺ monocytes and CD11b⁺Ly6G⁺ neutrophils in Batf3⁻/⁻ gWAT. **(H-I)** Immunofluorescence (IF) staining and quantification of myeloperoxidase (MPO, red; Nuclei, blue) confirming increased neutrophil infiltration in gWAT of BATF3⁻/⁻ mice. Scale bar, 100 μm. **(J)** MCP1 concentrations in gWAT were determined by ELISA. Increased MCP1 production in Batf3⁻/⁻ mice supports the elevated recruitment of CD11b⁺Ly6C⁺ monocytes observed in adipose tissue. Mice were fed a normal chow diet for 16 weeks. Data represent mean ± SD. *P < 0.05, **P < 0.01, ***P < 0.001 vs. WT.

To investigate the baseline phenotype associated with BATF3 deficiency, BATF3 knockout mice (Batf3⁻/⁻, Fig. S1A) were used to evaluate body weight, adipose tissue weight, liver weight, and related immune cell profiles in mice fed a normal chow diet. Compared to wild-type (WT) controls, Batf3⁻/⁻ mice displayed significantly increased body weight (Fig. 1A) and enlarged gonadal white adipose tissue (gWAT) mass (Fig. 1B) as well as gWAT/body weight ratio (Fig. 1C), suggesting that an obesity phenotype can be induced under normal conditions, although serum free fatty acids (FFA) showed no overt difference between genotypes (Fig. 1D). Since BATF3 plays a key role in shaping CD8α^+^ dendritic cells (cDC1s), which are vital for mounting robust inflammatory and immune responses 14, 15. We thought that BATF3 deficiency may impact the inflammatory response in adipose tissue. Interestingly, histological analysis of gWAT revealed that the number of crown-like structures (CLSs, black arrow), a key phenomenon of macrophage infiltration and dead adipocytes 8, was increased in Batf3⁻/⁻mice compared to WT mice (Fig. 1E), indicating ongoing adipose tissue inflammation. In parallel, flow cytometric analysis of immune cells in gWAT showed elevated infiltration of CD11b⁺Ly6C⁺ inflammatory monocytes and CD11b⁺Ly6G⁺ neutrophils in Batf3⁻/⁻ mice compared to WT mice (Fig. 1F and 1G). Additionally, immunofluorescence (IF) staining for myeloperoxidase (MPO, a neutrophil marker) and F4/80 (a macrophage marker), along with MCP1 levels, demonstrated elevated adipose tissue neutrophil infiltration and inflammation in Batf3⁻/⁻ mice (Fig. 1H-J, Fig. S1B), suggesting that BATF3 deficiency promotes adipose tissue immune activation under a basal metabolic condition. These findings indicate that BATF3 plays a protective role in maintaining immune homeostasis in adipose tissue under normal conditions.

**Figure 2:**
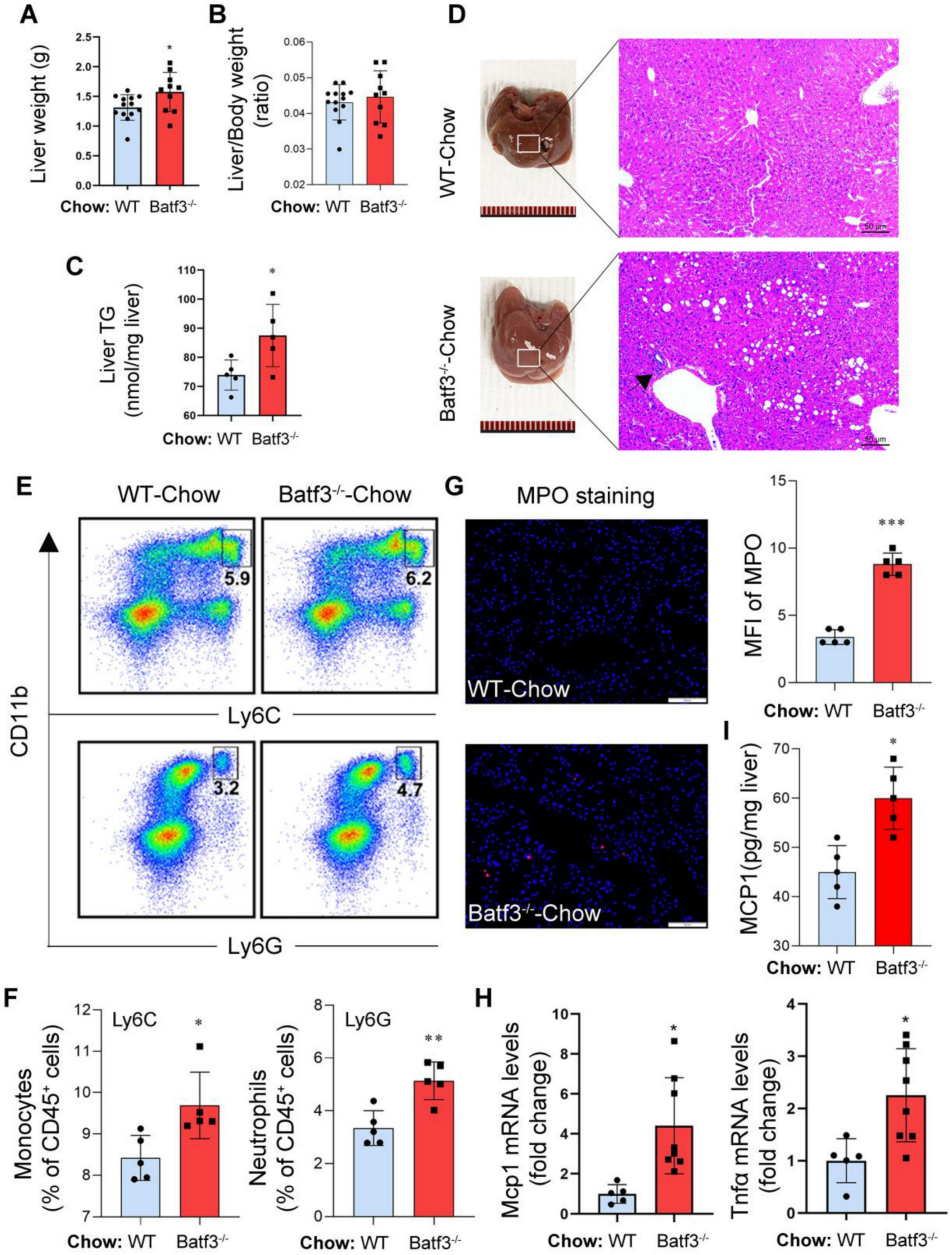
** BATF3 deficiency promotes hepatic steatosis and inflammation under normal chow diet conditions. (A-C)** Liver weight, liver/body weight ratio, and hepatic triglyceride (TG) content relative to WT. **(D)** H&E staining showed extensive lipid droplet deposition and inflammatory cell infiltration (black arrow) in the liver of Batf3⁻/⁻ mice. Scale bar, 50 μm. **(E-F)** Flow cytometry revealed increased CD11b⁺Ly6C⁺ monocytes and CD11b⁺Ly6G⁺ neutrophils in the liver of Batf3⁻/⁻ mice. **(G)** IF staining and quantification of myeloperoxidase (MPO, red; Nuclei, blue) in the liver of Batf3⁻/⁻ mice. Scale bar, 100 μm. **(H)** qPCR analysis of pro-inflammatory mediators Tnfα and Mcp1 in the liver of Batf3⁻/⁻ mice. **(I)** Hepatic MCP1 protein levels were quantified by ELISA. Increased MCP1 production in Batf3⁻/⁻ mice is consistent with enhanced recruitment of CD11b⁺Ly6C⁺ monocytes to the liver. Mice were fed a normal chow diet for 16 weeks. Data represent mean ± SD. *P < 0.05, **P < 0.01, ***P < 0.001 vs. WT.

Next, to assess whether BATF3 plays a role in maintaining hepatic metabolic and immune homeostasis under physiological conditions, we analyzed liver histology and immune profiles in Chow-fed mice. Batf3⁻/⁻ mice exhibited increased liver weight (Fig. 2A) without a change in liver/body weight ratio (Fig.2B), and elevated hepatic triglyceride (TG) content (Fig. 2C) compared to WT mice, suggesting spontaneous hepatic lipid accumulation. Histological staining revealed marked micro- and macrovesicular lipid droplet accumulation and increased inflammatory cell infiltration (black arrow) in Batf3⁻/⁻ mouse liver (Fig. 2D). Similarly, flow cytometric analysis showed higher frequencies of CD11b⁺Ly6C⁺ monocytes and CD11b⁺Ly6G⁺ neutrophils in the liver of Batf3⁻/⁻ mice (Fig. 2E and 2F), and IF for MPO confirmed increased neutrophil infiltration (Fig. 2G). In parallel, expression levels of key pro-inflammatory cytokines and chemokine, including Tnfα and Mcp1, were significantly upregulated (Fig. 2H and 2I), indicating an activated inflammatory state in Batf3⁻/⁻ mice. These findings suggest that BATF3 is required to suppress low-grade hepatic inflammation and lipid accumulation under normal conditions.

### BATF3 deficiency promotes adipocyte lipolysis and inflammation under high-fat diet feeding

**Figure 3:**
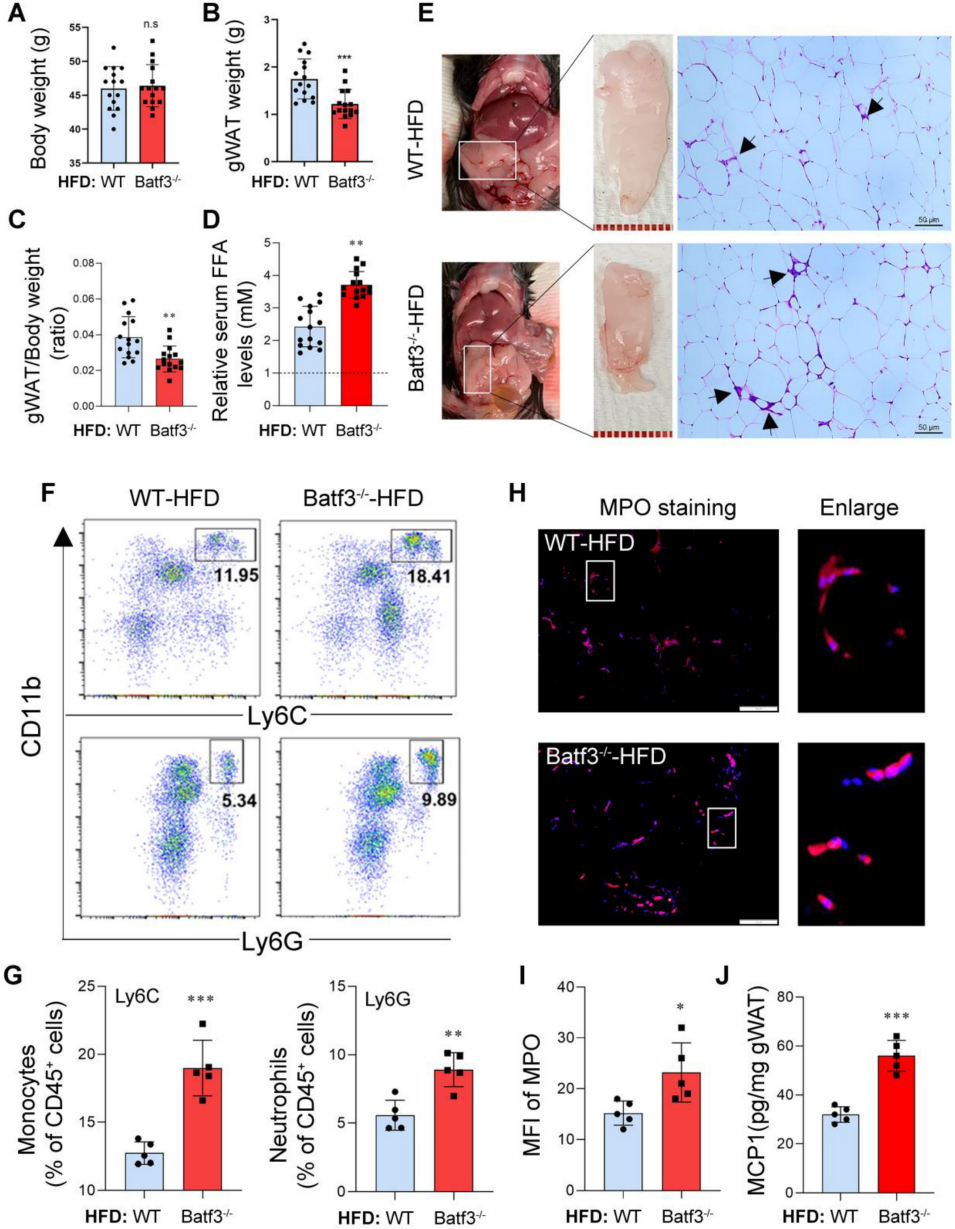
** BATF3 deficiency aggravates adipose tissue inflammation under high-fat diet feeding. (A-C)** Body weight, gWAT mass, and gWAT/body weight ratio in Batf3⁻/⁻ mice compared to WT under high-fat diet (HFD) feeding. **(D)** Circulating free fatty acid (FFA) levels were markedly increased in Batf3⁻/⁻ mice following HFD feeding. WT-Chow was used as the reference group and set to a value of 1. **(E)** H&E staining showed increased CLS formation (black arrows), reflecting adipocyte death and macrophage infiltration. **(F-G)** Flow cytometry revealed greater accumulation of inflammatory monocytes and neutrophils in the gWAT of Batf3⁻/⁻ mice. **(H-I)** IF staining and quantification of myeloperoxidase (MPO, red; Nuclei, blue) in gWAT of Batf3⁻/⁻ mice. **(J)** MCP1 concentrations in gWAT were determined by ELISA. MCP1 production was further increased in Batf3⁻/⁻ mice following HFD feeding, consistent with enhanced recruitment of CD11b⁺Ly6C⁺ monocytes to adipose tissue. Mice were fed a high-fat diet for 16 weeks. Data represent mean ± SD. n.s means no significant difference, *P < 0.05, **P < 0.01, ***P < 0.001 vs. WT.

Building on these baseline findings, we next asked whether BATF3 deficiency would further exacerbate metabolic and inflammatory disturbances under conditions of dietary stress. Interestingly, under high-fat diet (HFD) feeding, Batf3⁻/⁻ mice showed no significant difference in overall body weight compared to WT mice (Fig. 3A). In contrast, gWAT mass and ratio of gWAT/body weight were significantly reduced in Batf3⁻/⁻ mice (Fig. 3B and 3C). This observation differs from the chow-fed condition, where BATF3 deficiency promoted gWAT expansion, potentially reflecting enhanced adipocyte lipolysis (Fig. 3D) or inflammation-driven tissue remodeling in HFD-fed Batf3⁻/⁻ mice. Furthermore, histological analysis of gWAT revealed that high-fat diet feeding increased the number of crown-like structures (CLSs, black arrows) in WT mice, and this increase was further elevated in Batf3⁻/⁻ mice (Fig. 3E), indicating elevated macrophage infiltration and adipocyte death. In parallel, flow cytometric analysis of immune cells in gWAT showed increased infiltration of CD11b⁺Ly6C⁺ monocytes and CD11b⁺Ly6G⁺ neutrophils in Batf3⁻/⁻ mice (Fig. 3F and 3G). MPO and F4/80 immunostaining as well as MCP1 levels further confirmed enhanced hepatic neutrophil infiltration and inflammation (Fig. 3H-J, Fig. S1B), reinforcing a pro-inflammatory adipose tissue immune phenotype. These results indicate that BATF3 deficiency promotes adipose tissue inflammation and adipocyte lipolysis under high-fat diet conditions.

### BATF3 deficiency exacerbates hepatic steatosis and inflammation under high-fat diet conditions

**Figure 4:**
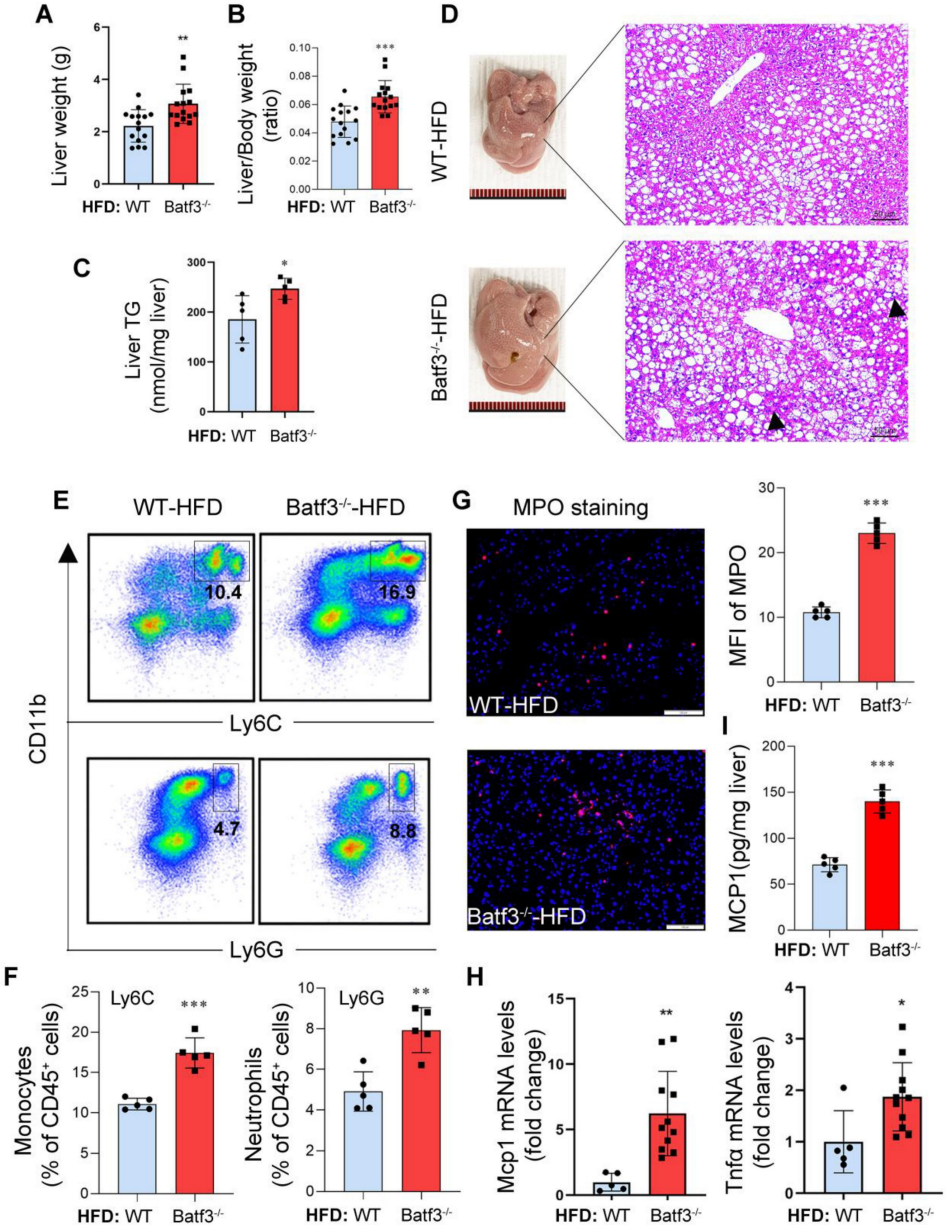
** BATF3 deficiency exacerbates hepatic steatosis and inflammation under high-fat diet feeding. (A-C)** Liver weight, liver/body weight ratio, and hepatic triglyceride (TG) content compared with WT controls following HFD feeding, indicating aggravated hepatic steatosis. **(D)** H&E staining revealed extensive micro- and macrovesicular lipid droplet deposition accompanied by prominent immune cell infiltration (black arrows) in the liver of Batf3⁻/⁻ mice. Scale bar, 50 μm. **(E-F)** Flow cytometric analysis showed a higher frequency of CD11b⁺Ly6C⁺ inflammatory monocytes and CD11b⁺Ly6G⁺ neutrophils in the liver of Batf3⁻/⁻ mice compared with WT mice. **(G)** IF staining and quantification of myeloperoxidase (MPO, red; Nuclei, blue) in the liver of Batf3⁻/⁻ mice. Scale bar, 100 μm. **(H)** qPCR analysis of pro-inflammatory mediators Tnfα and Mcp1 in the liver of Batf3⁻/⁻ mice. **(I)** Hepatic MCP1 protein levels were quantified by ELISA. Elevated MCP1 production in Batf3⁻/⁻ mice is consistent with enhanced recruitment of CD11b⁺Ly6C⁺ monocytes to the liver under HFD conditions. Mice were fed a high-fat diet for 16 weeks. Data represent mean ± SD. *P < 0.05, **P < 0.01, ***P < 0.001 vs. WT.

Next, we investigated whether fat degraded in adipose tissue could be transported to the liver, leading to ectopic deposition and thereby exacerbating hepatic steatosis. As expected, liver weight (Fig. 4A), liver/body weight ratio (Fig. 4B), and hepatic triglyceride (TG) content (Fig. 4C) were significantly increased after HFD feeding in Batf3⁻/⁻ mice. Histological analysis also revealed extensive lipid droplet accumulation and inflammatory cell infiltration in the liver of Batf3⁻/⁻ mouse (Fig. 4D), which is consistent with the report of aggravated steatohepatitis 18. Furthermore, flow cytometric analysis showed enhanced infiltration of CD11b⁺Ly6C⁺ inflammatory monocytes and CD11b⁺Ly6G⁺ neutrophils in the liver of Batf3⁻/⁻ mice (Fig. 4E and 4F), while IF staining for myeloperoxidase (MPO) confirmed a substantial increase in hepatic neutrophil accumulation (Fig. 4G). Additionally, hepatic mRNA expression of Tnfα and Mcp1, as well as protein levels of MCP1, were significantly elevated in Batf3⁻/⁻ mouse (Fig. 4H and 4I), indicating an amplified inflammatory response. Together, these results demonstrate that BATF3 deficiency aggravates hepatic lipid accumulation and immune activation in HFD-induced metabolic disturbances, when adipose tissue lipolysis.

### BATF3 deficiency impairs hepatic lipid homeostasis, and exacerbates oxidative stress

To better understand the mechanisms underlying aggravated steatosis, we next analyzed pathways involved in hepatic lipid handling. BATF3 deficiency did not further enhance the expression of most lipogenic genes under HFD feeding (Fig. S2A), indicating that *de novo* lipogenesis was unlikely to account for the increased hepatic TG accumulation. Instead, we observed upregulation of PPARγ (Fig. S2B-D), a nuclear receptor that promotes lipid uptake and storage programs and has been implicated in hepatic steatosis and insulin resistance in metabolic liver diseases 20.

Indeed, BATF3 deficiency markedly increased the expression of CD36, whereas Fabp5, Fatp2, and Fatp5 remained unchanged (Fig. S2A-D), suggesting that enhanced uptake of circulating fatty acids does not lead to significant alteration in intracellular fatty acid transport. Consistent with increased fatty acid influx, BATF3 deficiency further elevated the expression of ACSL1 and ACOX1 (Fig. S2B-F). Both the ACSL1 and ACOX1 are involved in conversion of related free fatty acids into acyl-CoAs, which serve as substrates for both β-oxidation and glycerolipid synthesis 21, 22. Therefore, although *Dgat1/2* levels were unchanged, enhanced substrate availability may still drive triglyceride synthesis under conditions of excessive fatty acid influx. In addition, BATF3 deficiency impaired hepatic VLDL-TG secretion (Fig. S2G), which would further favor intracellular triglyceride retention and thereby exacerbate hepatic TG accumulation.

Extensive evidence in MASLD indicates that excessive fatty acid influx can overwhelm mitochondrial/peroxisomal β-oxidation, leading to increased reactive oxygen species (ROS) production and oxidative stress, one of the central pathological features of disease progression 23. In parallel, surplus fatty acids, particularly saturated species, can activate NADPH oxidase (NOX), further amplifying ROS generation and promoting inflammation and insulin resistance during MASLD progression 23, 24. Consistent with these mechanisms, we found that the expression of NOX2, but not NOX4, was significantly increased in Batf3⁻/⁻ mice under HFD feeding (Fig. S2B and S3A-B). Moreover, IHC staining and Western blotting revealed upregulation of C/EBP homologous protein (CHOP) and 4-hydroxynonenal (4-HNE) (Fig. S3C-G), confirming enhanced ER stress and oxidative stress under these conditions.

### BATF3 deficiency exacerbates insulin resistance in the liver and adipose tissue

**Figure 5:**
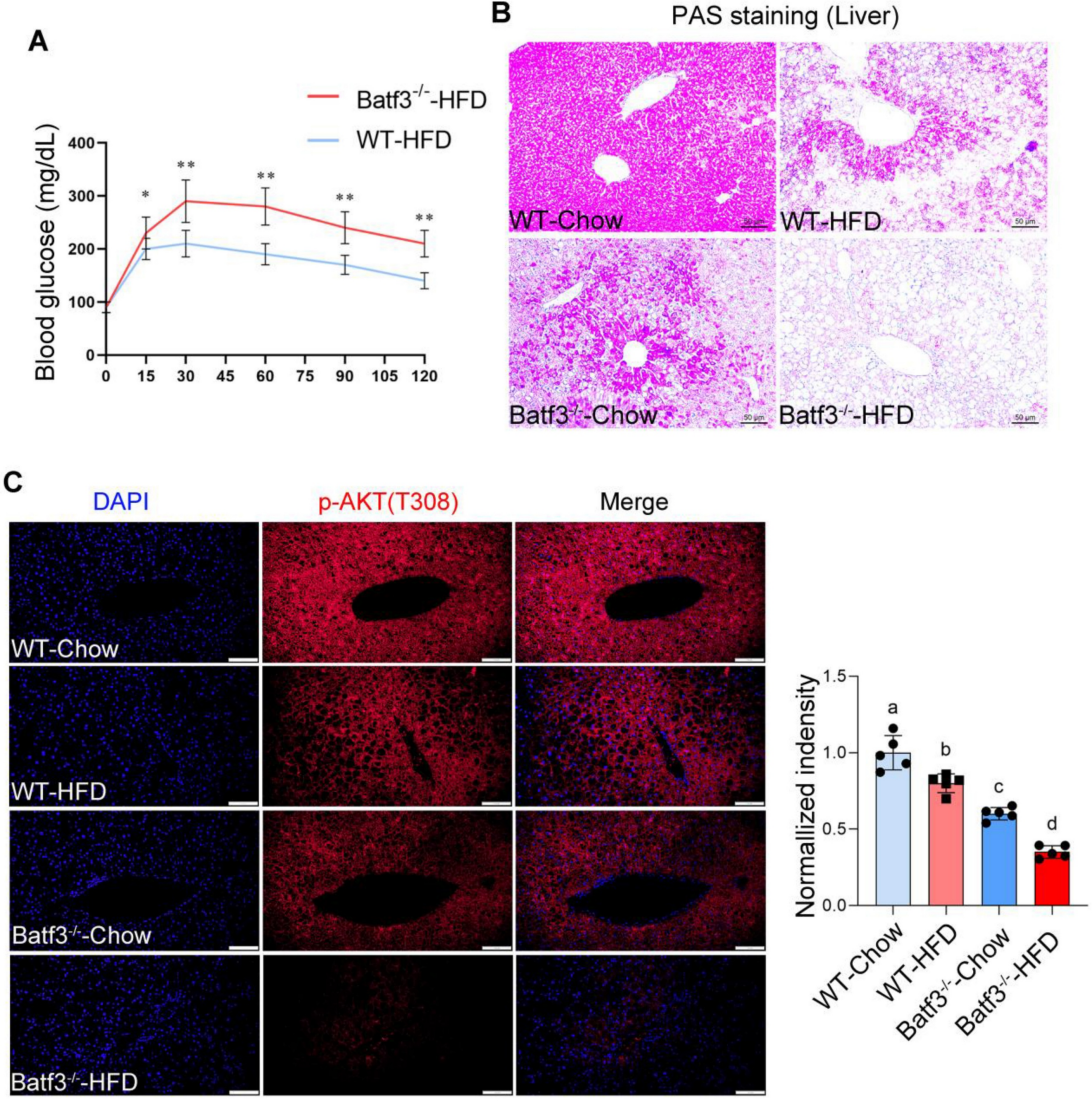
** BATF3 deficiency exacerbates systemic and hepatic insulin resistance under high-fat diet feeding. (A)** Oral glucose tolerance test (OGTT) analysis demonstrated that Batf3⁻/⁻ mice displayed markedly impaired glucose clearance compared with WT controls, indicating systemic insulin resistance. **(B)** Periodic acid-Schiff (PAS) staining of liver sections revealed significantly reduced glycogen storage in Batf3⁻/⁻ mice. Scale bar, 50 μm. **(C)** IF staining for phosphorylated AKT (p-AKT) showed diminished insulin signaling activity in the livers of Batf3⁻/⁻ mice compared with WT controls. Scale bar, 100 μm. Mice were fed a high-fat diet for 16 weeks. Data represent mean ± SD. Different letters represent significant differences. *P < 0.05, **P < 0.01, ***P < 0.001 vs. WT.

Insulin resistance (IR) is recognized as a central mechanism in the initiation and progression of MASLD 25, characterized by impaired insulin sensitivity in the liver, adipose tissue, and peripheral organs 26. To determine whether BATF3 modulates systemic and tissue-specific insulin sensitivity, we first performed OGTT in HFD-fed WT and Batf3⁻/⁻ mice. BATF3 deletion markedly impaired glucose tolerance and elevated fasting blood glucose levels (Fig. 5A). Since hepatic IR is not only associated with hepatic TG accumulation but also with altered glucose metabolism 27. We next examined glycogen storage and genes involved in gluconeogenesis and glycolysis. PAS staining revealed that BATF3 deficiency significantly reduced hepatic glycogen content (Fig. 5B). However, no changes in gluconeogenic genes (G6pc, Pck1) or the glucose transporter Slc2a2 were observed by qPCR analysis, whereas glycolysis-related genes (Gck, Hk1) were significantly increased (Fig. S4A), suggesting inflammation-driven metabolic reprogramming. Consistently, IF staining demonstrated that HFD reduced hepatic p-AKT expression, which was further exacerbated in Batf3⁻/⁻ mice (Fig. 5C), supporting a critical role of BATF3 in maintaining hepatic insulin signaling 28, 29.

**Figure 6:**
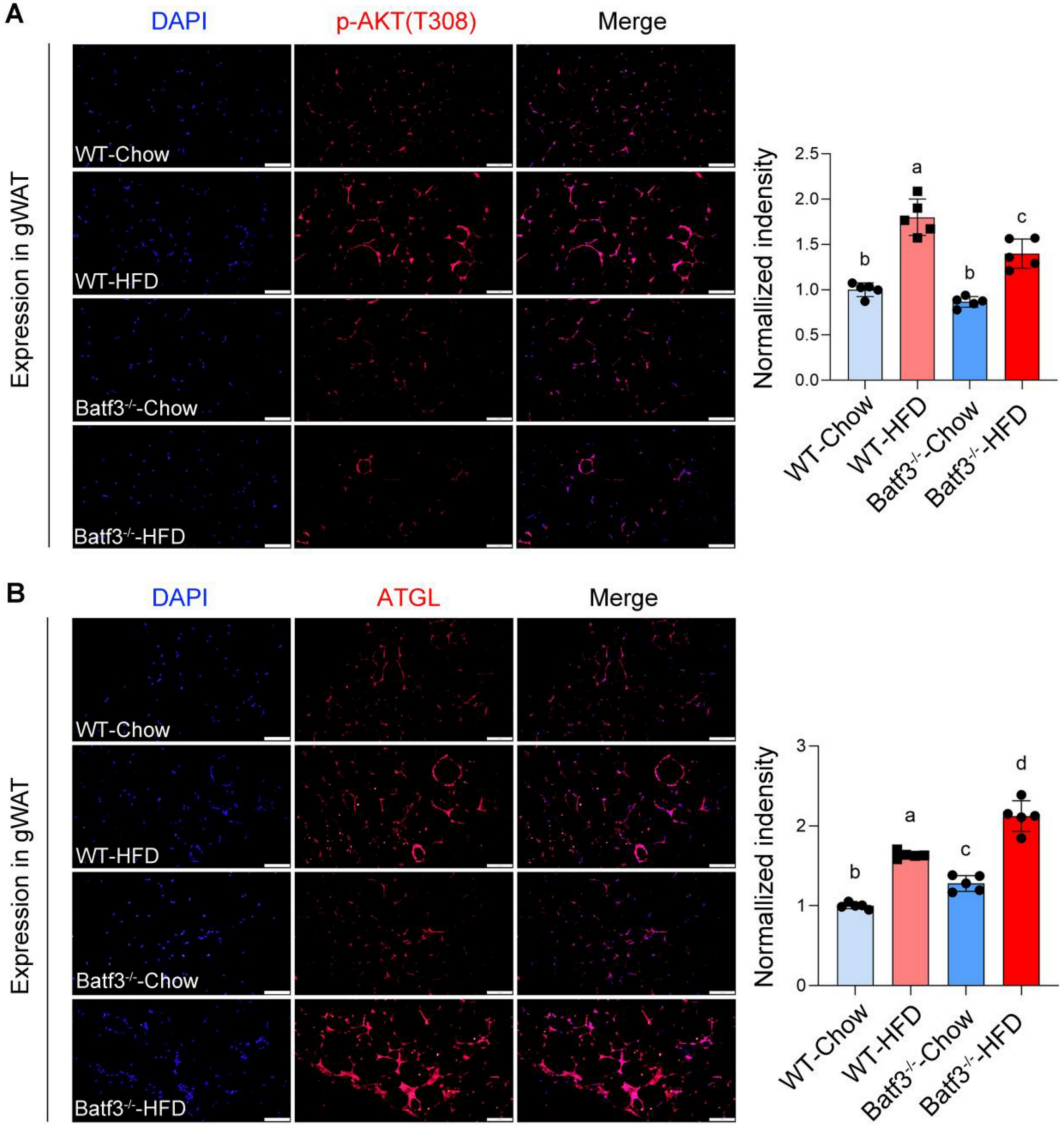
** BATF3 deficiency impairs adipose insulin signaling and enhances lipolysis under HFD feeding. (A)** IF staining for phosphorylated AKT (p-AKT) was performed on gonadal white adipose tissue (gWAT) sections from WT and Batf3⁻/⁻ mice fed a high-fat diet (HFD). **(B)** ATGL, a key lipolytic enzyme that hydrolyzes stored triglycerides into free fatty acids, was examined by IF staining of gWAT sections. All scale bar, 100 μm. Mice were fed a high-fat diet for 16 weeks. Data represent mean ± SD. Different letters represent significant differences.

Because insulin-AKT signaling also regulates lipid metabolism in adipose tissue by promoting lipogenesis and suppressing lipolysis 30, 31. We next evaluated this signaling in adipose tissue. IF staining revealed that HFD feeding increased p-AKT expression in adipose tissue, consistent with enhanced lipid storage, whereas BATF3 deficiency markedly blunted this response (Fig. 6A). Reduced AKT activation was accompanied by increased expression of ATGL (Fig. 6B), a key lipolytic enzyme that hydrolyzes lipid droplet-derived TG into FFA 32. These findings provide a mechanistic basis for the observed reduction in gWAT weight in Batf3⁻/⁻ mice under HFD feeding (Fig. 3B). Together, these results demonstrate that BATF3 is required for preserving systemic insulin sensitivity, protecting hepatic insulin signaling, and restraining adipose lipolysis in response to HFD.

### BATF3 deficiency promotes hepatic fibrosis under a high-fat diet condition

**Figure 7:**
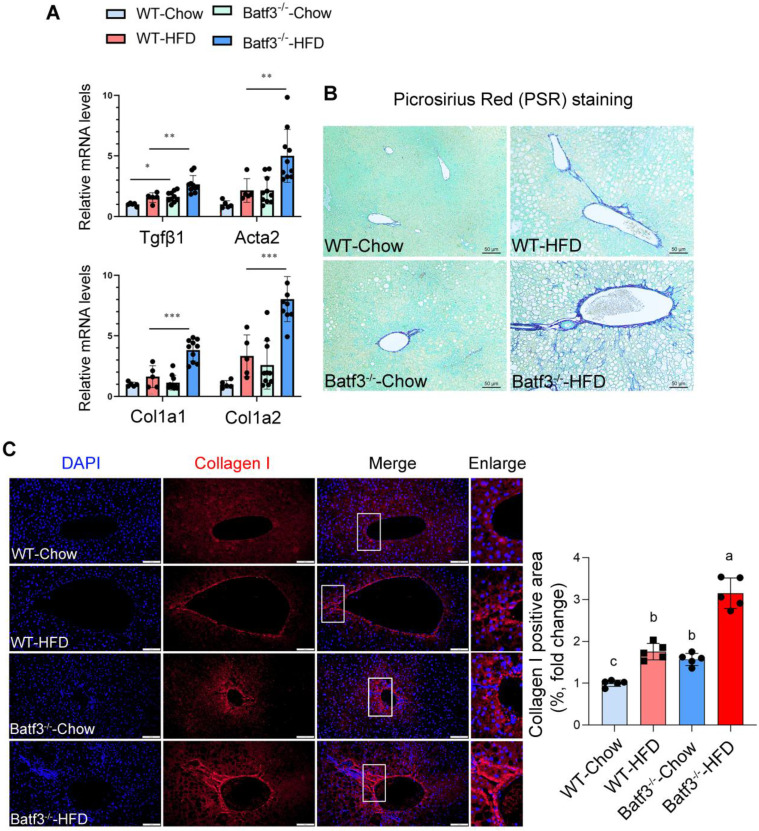
** BATF3 deficiency promotes hepatic fibrosis under high-fat diet feeding. (A)** qPCR analysis of hepatic expression of fibrosis-related genes, including *Tgfβ1*, *Acta2*, *Col1a1*, and *Col1a2* in WT and Batf3⁻/⁻ mice. **(B)** Picrosirius red (PSR) staining was performed on liver sections to visualize collagen deposition. Batf3⁻/⁻ mice displayed markedly increased collagen accumulation compared with WT controls. Scale bar, 50 μm. **(C)** IF staining of collagen I further confirmed excessive collagen fiber formation in the liver of Batf3⁻/⁻ mice after HFD challenge. Scale bar, 100 μm. Mice were fed a high-fat diet for 16 weeks. Data represent mean ± SD. Different letters represent significant differences. *P < 0.05, **P < 0.01, ***P < 0.001 vs. WT.

Chronic hepatic inflammation is a well-established driver of fibrogenesis 33, 34, as pro-inflammatory cytokines and immune cell infiltration both trigger hepatic stellate cell activation 35, 36. Although a previous report indicated that BATF3 deficiency did not influence fibrosis progression under high-sucrose or methionine- and choline-deficient diet challenges 18, its role in the development of fibrosis in response to HFD remained unclear. Given our observation of exacerbated hepatic inflammation in Batf3⁻/⁻ mice under HFD conditions (Fig. 4D-I), we hypothesized that BATF3 loss might accelerate fibrogenic progression. Indeed, fibrotic markers including transforming growth factor beta 1 (Tgfβ1), alpha smooth muscle actin (Acta2), and type I collagen isoforms (Col1a1, Col1a2) were markedly upregulated in the liver of Batf3⁻/⁻ mouse compared with WT controls (Fig. 7A). Consistent with these transcriptional changes, histological analysis revealed significantly increased collagen deposition, as demonstrated by Picrosirius Red staining (Fig. 7B) and collagen I immunofluorescence staining (Fig. 7C). Together, these findings indicate that BATF3 deficiency enhances hepatic fibrogenesis in HFD-induced steatosis, at least partially through amplification of inflammatory signaling.

### BATF3 deficiency reduces hepatokine ANGPTL8 expression in the liver and adipose tissue

**Figure 8:**
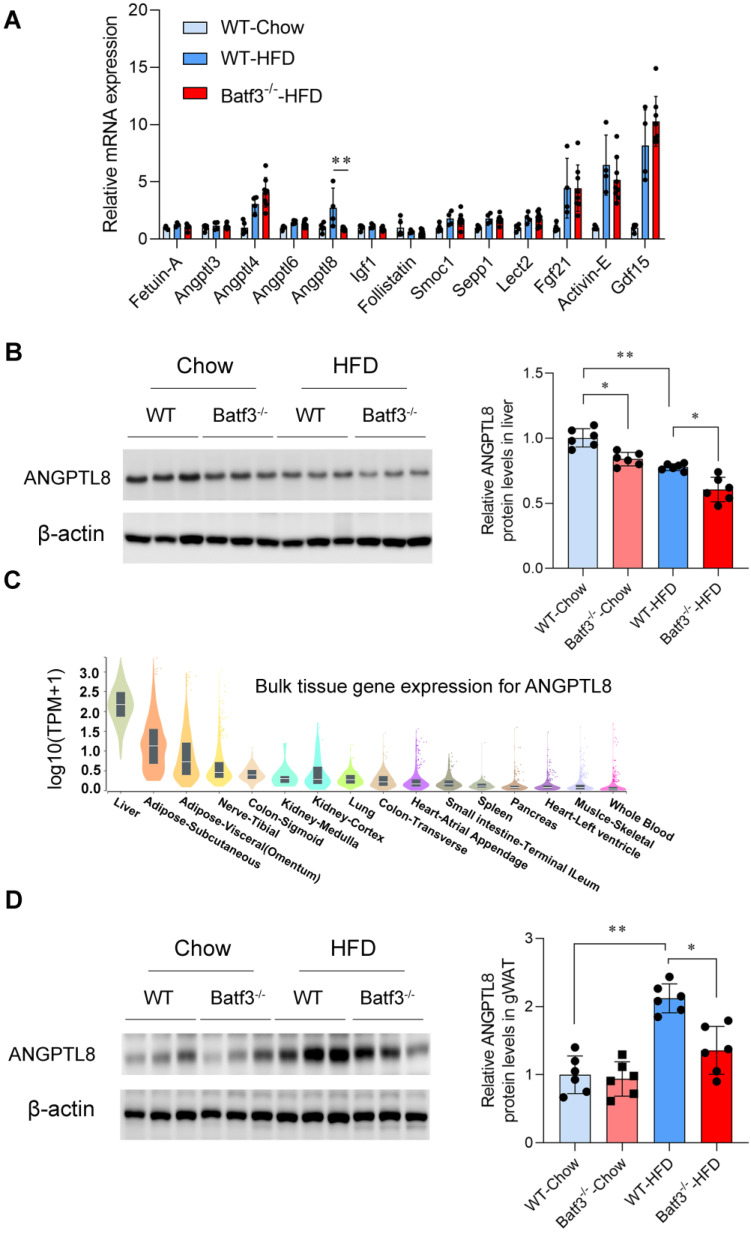
** BATF3 deficiency selectively reduces hepatokine ANGPTL8 expression. (A)** qPCR analysis of hepatic expression of multiple hepatokines in WT and Batf3⁻/⁻ mice after HFD feeding. Gene expression was quantified by qPCR and normalized to housekeeping genes using the ΔΔCt method. WT-Chow was used as the reference group and set to a value of 1. **(B)** Western blot analysis confirmed markedly reduced ANGPTL8 protein levels in the liver of Batf3⁻/⁻ mice compared with WT controls under HFD feeding, n = 6/group. **(C)** Human tissue distribution of ANGPTL8 transcripts was analyzed using the GTEx database, expressed as transcripts per million (TPM). ANGPTL8 was abundantly expressed in both liver and adipose tissue. **(D)** Western blot analysis confirmed markedly reduced ANGPTL8 protein levels in the adipose tissue of Batf3⁻/⁻ mice compared with WT controls under HFD feeding, n = 6/group. All scale bar, 100 μm. Mice were fed a high-fat diet for 16 weeks. Data represent mean ± SD. *P < 0.05, **P < 0.01vs. WT.

With the observation of adipose tissue lipolysis and aggravated hepatic steatosis in Batf3⁻/⁻ mice, we next examined whether hepatocyte-derived factors could mediate the crosstalk between immune dysregulation and systemic metabolic disturbances. Hepatokines are hormone-like proteins secreted by hepatocytes that regulate inter-organ communication, and accumulating evidence implicates them in the development of insulin resistance and fatty liver disease 9, 37. We therefore examined hepatic expression of major hepatokines in WT and Batf3⁻/⁻ mice under HFD feeding. In WT mice, HFD decreased Follistatin expression while markedly increasing Angptl4, Angptl8, Fgf21, Activin-E, and Gdf15. In contrast, BATF3 deficiency selectively abolished the HFD-induced upregulation of Angptl8, whereas the expression of other hepatokines remained largely unchanged (Fig. 8A). This selective reduction of ANGPTL8 was further confirmed at the protein level by Western blot analysis (Fig. 8B) and IF staining (Fig. S5A), indicating that ANGPTL8 expression is impaired in the setting of BATF3 deficiency under metabolic stress conditions.

It has been reported that ANGPTL8 regulates systemic lipid and glucose metabolism through its effects on both hepatocytes and peripheral tissues, although other studies have produced divergent results regarding its metabolic roles 11, 12, 38. In our current study, we analyzed human ANGPTL8 transcript distribution using the Genotype-Tissue Expression (GTEx) database. Consistent with earlier evidence 39, ANGPTL8 was not only found in the liver but also expressed in adipose tissue (Fig. 8C, Fig. S5B), suggesting a potential tissue-specific heterogeneity. Supporting this observation, Western blot (Fig. 8D) and IF staining analysis (Fig. S5C) demonstrated that ANGPTL8 expression in gWAT was low under chow conditions but markedly induced by HFD feeding. Importantly, this HFD-induced increase was attenuated in Batf3⁻/⁻ mice. Collectively, these findings indicate that BATF3 is required for proper expression of ANGPTL8 in both liver and adipose tissue. Selective downregulation of ANGPTL8 in Batf3⁻/⁻ mice may contribute to enhanced adipose lipid mobilization and impaired hepatic lipid handling, thereby providing a mechanistic link between immune dysregulation and systemic metabolic imbalance.

### ANGPTL8 treatment modulates lipid metabolism and fibrogenic responses *in vitro*

BATF3 is largely restricted to the dendritic cell lineage and functions as a lineage-defining transcription factor for cDC1 40. Accordingly, the metabolic abnormalities observed in Batf3⁻/⁻ mice likely arise from immune-mediated perturbations rather than cell-autonomous defects in hepatocytes or adipocytes. We therefore sought to define the upstream inflammatory signals governing ANGPTL8 expression and to delineate the functional consequences of ANGPTL8 restoration across metabolically relevant cell types.

In HepG2 hepatocytes, FFA treatment did not alter ANGPTL8 expression, whereas TNFα stimulation markedly reduced ANGPTL8 levels (Fig. S6A), indicating that inflammatory signaling—rather than lipid overload *per se*—directly suppresses ANGPTL8. A similar response was observed in differentiated 3T3-L1 adipocytes, where TNFα significantly downregulated ANGPTL8 expression (Fig. S6B), suggesting a conserved inflammatory repression mechanism in both hepatic and adipose compartments. To establish causality, we silenced TNF receptor 1 (TNF-R1) in HepG2 cells using siRNA targeting *Tnfrsf1a* (Fig. S6C). TNF-R1 knockdown significantly attenuated TNFα-induced downregulation of ANGPTL8 (Fig. S6D), suggesting TNFα suppresses ANGPTL8 expression through a TNF-R1-dependent signaling pathway in hepatocytes.

**Figure 9:**
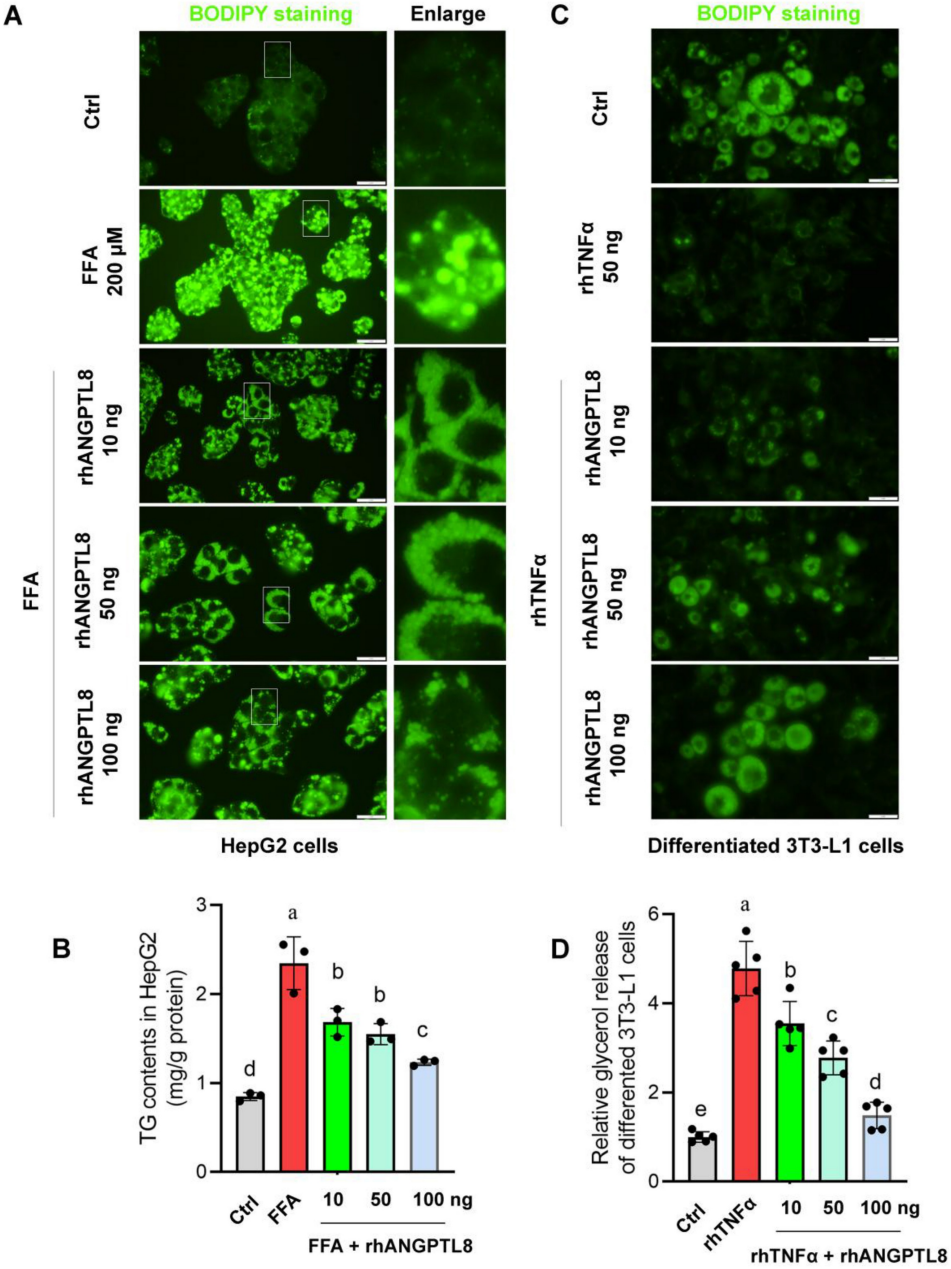
** ANGPTL8 treatment mitigates hepatocyte lipid accumulation, and adipocyte lipolysis. (A)** BODIPY staining of HepG2 hepatocytes treated with free fatty acids (FFA) in the presence or absence of recombinant ANGPTL8, showing reduced lipid droplet accumulation with ANGPTL8 treatment. Scale bar, 2 mm. **(B)** Quantification of intracellular triglyceride content in HepG2 cells confirmed that ANGPTL8 significantly reduced FFA-induced triglyceride accumulation. **(C)** BODIPY staining of differentiated 3T3-L1 adipocytes exposed to TNFα with or without recombinant ANGPTL8. ANGPTL8 treatment decreased TNFα-induced lipid droplet breakdown. Scale bar, 2 mm. **(D)** Glycerol release assay in 3T3-L1 adipocytes demonstrated that ANGPTL8 suppressed TNFα-induced lipolysis. Data represent mean ± SD. Different letters represent significant differences.

**Figure 10:**
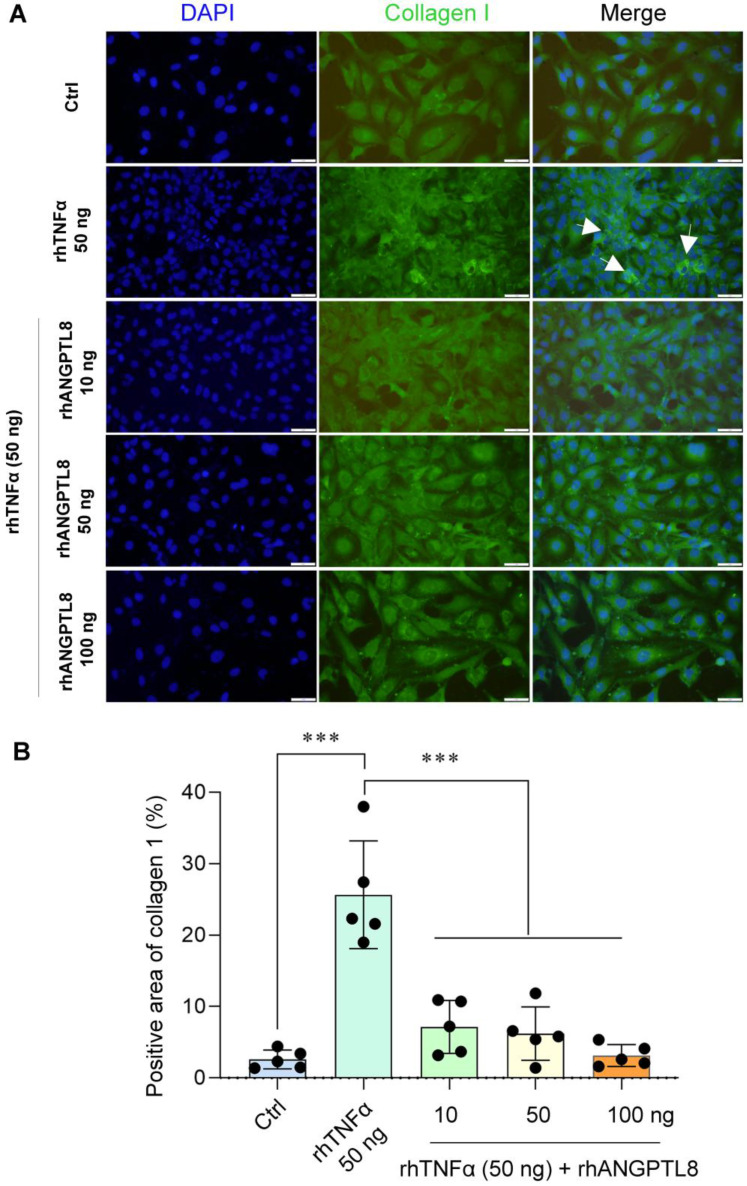
** ANGPTL8 treatment mitigates hepatic stellate cell activation. (A)** IF staining of collagen I deposition in LX-2 stellate cells stimulated with TNFα in the presence or absence of ANGPTL8, showing attenuated stellate cell activation following ANGPTL8 treatment (white arrow points to collagen I deposition). Scale bar, 2 mm. **(B)** Quantification of Collagen I-positive area in LX-2 cells under the indicated treatment conditions. The percentage of collagen I-positive area was calculated from five independent microscopic fields per group (n = 5) using uniform thresholding criteria. Data are presented as mean ± SD. ***P < 0.001 versus control (Ctrl) group or TNFα-treated group.

Based on this finding, we next examined whether restoring ANGPTL8 could counteract inflammation- and lipid-driven metabolic dysfunction. In HepG2 cells, recombinant ANGPTL8 significantly reduced FFA-induced triglyceride (TG) accumulation (Fig. 9A-B), demonstrating a direct lipid-lowering effect in hepatocytes. In differentiated 3T3-L1 adipocytes, ANGPTL8 suppressed TNFα-induced lipolysis, as evidenced by reduced glycerol release (Fig. 9C-D). Moreover, in LX-2 hepatic stellate cells, ANGPTL8 attenuated TNFα-induced activation, reflected by decreased collagen I deposition (Fig. 10A-B), indicating an anti-fibrogenic effect.

To further define the molecular basis of ANGPTL8-mediated lipid regulation, we evaluated key proteins involved in triglyceride mobilization and export. In HepG2 cells, ANGPTL8 markedly blunted FFA-induced upregulation of adipose triglyceride lipase (ATGL), a rate-limiting enzyme for triglyceride hydrolysis (Fig. S7A-B). Notably, ANGPTL8 also mitigated the FFA-induced reduction of ApoB, suggesting partial restoration of ApoB-dependent lipoprotein assembly and triglyceride export. In contrast, ANGPTL8 did not affect DGAT1 expression, arguing against an effect on triglyceride synthesis/esterification. Consistently, in differentiated 3T3-L1 adipocytes, ANGPTL8 reduced TNFα-induced ATGL expression without altering DGAT1 levels (Fig. S7C-D), supporting a conserved role in restraining inflammation-driven lipolysis.

Together, these data support a model in which inflammatory suppression of ANGPTL8 amplifies metabolic stress by simultaneously enhancing ATGL-mediated triglyceride mobilization and compromising ApoB-dependent lipid export, thereby promoting maladaptive lipid flux and hepatic lipid overload. Conversely, ANGPTL8 restoration exerts coordinated multi-tissue protective effects—limiting hepatocyte TG accumulation, restraining adipose lipolysis, and suppressing stellate cell activation, thereby interrupting the pathogenic feed-forward cycle linking lipid flux, inflammation, and progression from steatosis to fibrosis.

## 4. Discussion

In the current study, we identified BATF3 as a key regulator of adipose-liver crosstalk under high-fat diet challenge. Loss of BATF3 promoted adipose tissue lipolysis and inflammation, enhanced hepatic fatty acid uptake and lipid accumulation, aggravated oxidative stress and insulin resistance, and ultimately accelerated fibrogenesis. Mechanistically, BATF3 deficiency disrupted the normal induction of the hepatokine ANGPTL8 during HFD feeding, and *in vitro* assays showed that ANGPTL8 restrains hepatocyte triglyceride accumulation, suppresses adipocyte lipolysis, and limits stellate cell activation. Together, these findings suggest that BATF3 maintains immune-metabolic homeostasis in part by sustaining hepatokine-mediated coordination between the liver and adipose tissue.

MASLD arises from the convergence of dysregulated lipid metabolism 41, insulin resistance 42, inflammation 43, and organ-to-organ communication 44, 45. While previous studies suggested that BATF3 deficiency does not significantly affect fibrosis progression in models such as high-sucrose or methionine-choline-deficient diets 18. Our findings reveal that under high-fat diet (HFD) feeding, BATF3 deficiency leads to pronounced fibrotic changes, including increased collagen deposition, stellate cell activation, and upregulation of profibrotic genes. This discrepancy likely reflects the distinct metabolic stress imposed by HFD, which enhances FFA influx to the liver from the adipose tissue and unmasks defects in immune-metabolic regulation. In our model, BATF3 deficiency impaired the insulin-AKT signaling in adipose tissue, increased ATGL expression, and promoted infiltration of inflammatory monocytes and neutrophils, thereby driving excessive lipolysis and FFA release. In the liver, this heightened FFA influx was compounded by reduced VLDL-TG secretion, resulting in lipid overload, oxidative stress, and impaired insulin signaling. These factors collectively created a permissive environment for hepatic inflammation and fibrogenesis.

In line with these findings, the exacerbated steatosis observed in HFD-fed Batf3⁻/⁻ mice was not driven by increased *de novo* lipogenesis, as most lipogenic genes remained unchanged. Rather, BATF3 deficiency disrupted hepatic lipid homeostasis at the level of fatty acid flux. Upregulation of PPARγ and CD36 indicates enhanced hepatic uptake of circulating fatty acids, likely derived from increased adipose tissue lipolysis. Concomitantly, increased expression of ACSL1 and ACOX1 reflects elevated intracellular fatty acid activation and channeling to fatty acid metabolic pathways in response to fatty acid oversupply. Because ACSL1 and ACOX1 are involved in the conversion of free fatty acids into acyl-CoAs, which serve as central intermediates for both β-oxidation and glycerolipid synthesis, their upregulation can expand the intracellular acyl-CoA pool under conditions of excessive fatty acid influx. Therefore, even without changes in Dgat1/2 expression, enhanced substrate availability may still drive triglyceride synthesis. Importantly, impaired VLDL-TG secretion further limits hepatic lipid export, thereby favoring intracellular triglyceride retention. Collectively, these alterations point to a state of dysregulated fatty acid throughput characterized by increased free fatty acids influx, expanded substrate availability, and defective export, ultimately resulting in hepatic TG accumulation. Excessive fatty acid flux is well recognized as a driver of oxidative stress in MASLD 23. When FFA delivery exceeds mitochondrial and peroxisomal handling capacity, oxidative pathways become overloaded, leading to increased reactive oxygen species production. In parallel, saturated fatty acids can activate NADPH oxidase, further amplifying ROS generation and inflammatory signaling. Consistent with this model, BATF3-deficient livers exhibited increased NOX2 expression together with elevated oxidative and ER stress markers, including CHOP and 4-HNE, suggesting that lipid-driven metabolic stress promotes a redox imbalance and therefore exacerbates hepatocellular injury and accelerates fibrogenic progression under HFD challenge.

Beyond these metabolic disturbances, our study highlights ANGPTL8 as a key hepatokine linking immune activation to lipid handling. We found that ANGPTL8 expression was selectively reduced in Batf3⁻/⁻ mice under HFD feeding. Notably, this downregulation was not a direct consequence of lipid overload, since FFA treatment alone did not suppress ANGPTL8 expression in hepatocytes. Instead, inflammatory signaling emerged as the major driver for ANGPTL8 suppression. Our additional *in vitro* data indicate that TNFα markedly suppresses ANGPTL8 expression in both hepatocytes and adipocytes, whereas FFA exposure alone has no significant effect. Moreover, silenced TNFR1 significantly attenuated TNFα-induced downregulation of ANGPTL8, identifying a TNFα-TNFR1 signaling axis as a key upstream regulator of ANGPTL8 under inflammatory conditions. These findings suggest that the reduced ANGPTL8 expression observed in Batf3⁻/⁻ mice is likely secondary to heightened inflammatory signaling rather than being directly driven by lipid/fatty acid accumulation. Given that BATF3 deficiency promotes immune activation and increases pro-inflammatory cytokine production, enhanced TNFα signaling may mechanistically link immune dysregulation to impaired hepatokine induction. Functionally, ANGPTL8 plays a critical role in maintaining lipid homeostasis and limiting fibrogenic activation. Consistent with previous findings that ANGPTL8 regulates triglyceride trafficking from the liver to peripheral tissues 46. The study showed that plasma triglyceride levels are lower in Angptl8^+/-^ mice, and mice lacking ANGPTL8 abolished the increased triglyceride delivery to adipose tissue after food intake 46. We observed that recombinant ANGPTL8 reduced FFA-induced triglyceride accumulation in hepatocytes, suppressed TNFα-induced lipolysis in adipocytes, and attenuated TNFα-driven collagen I deposition in hepatic stellate cells. These results suggest that loss of ANGPTL8 under inflammatory conditions may exacerbate the cycle of lipid retention, lipolysis, and stellate cell activation, thereby aggravating steatohepatitis and fibrosis.

It is also important to consider the tissue-specific contributions of ANGPTL8. Prior reports indicate that adipocyte-specific ANGPTL8 deletion promotes lipolysis but improves insulin sensitivity under high-fat/high-fructose diets 47, whereas hepatocyte-specific deficiency exacerbates insulin resistance 48. In our BATF3-deficient model, ANGPTL8 expression was reduced in both liver and adipose tissue. While loss of ANGPTL8 in adipose tissue was associated with enhanced lipolysis, it did not translate into improved systemic insulin sensitivity. Instead, reduced ANGPTL8 in the liver coincided with aggravated lipid accumulation and hepatic insulin resistance. Thus, our data support a tissue-specific role for ANGPTL8 in lipid metabolism. However, in the setting of systemic BATF3 deficiency, reduced ANGPTL8 levels are accompanied by worsening insulin resistance. This likely reflects the broader immune-metabolic dysregulation induced by BATF3 loss, which enhances adipose lipolysis and increases fatty acid flux to the liver. Under HFD feeding, elevated hepatic fatty acid influx combined with impaired VLDL export promotes lipid retention within hepatocytes. In this context, hepatic lipid overload likely represents a major driver of metabolic dysfunction, thereby contributing to systemic insulin resistance despite the complex, tissue-specific actions of ANGPTL8.

Of note, BATF3 expression is largely restricted to the dendritic cell lineage and functions as a lineage-defining transcription factor for cDC1 40. Therefore, the metabolic phenotype observed here is unlikely to reflect intrinsic defects in hepatocytes or adipocytes, but rather immune-mediated alterations in tissue homeostasis. Nevertheless, dendritic cells are broadly distributed across metabolically relevant organs, including liver, adipose tissue, and intestine, and may participate in inter-organ immune-metabolic communication 49. While our data support a predominant role of DC dysregulation within liver and adipose tissue under HFD challenge, the potential contribution of multi-organ immune coordination cannot be fully excluded. Further mechanistic studies will be required to define how BATF3-dependent immune networks integrate systemic metabolic regulation.

In summary, our work identifies BATF3 as an important regulator of hepatic and adipose tissue homeostasis and highlights the regulatory role of ANGPTL8 in mice with BATF3 deficiency. These findings not only provide mechanistic insight into how immune imbalance accelerates the progression from steatosis to fibrosis but also suggest that restoring BATF3-dependent pathways or modulating ANGPTL8 activity could represent potential therapeutic strategies against MASLD. Future studies should explore how BATF3-dependent immune cells activation shapes hepatokine networks, and whether tissue-specific ANGPTL8 delivery can counteract metabolic inflammation *in vivo*.

## Supplementary Material

Supplementary figures and tables.
