# Supplementary material for: Loss of BATF3 impairs adipose-liver homeostasis and accelerates the transition from steatosis to fibrosis in high-fat diet-fed mice
Source: Int J Biol Sci. 2026 Apr 16;22(9):4441–60. doi: 10.7150/ijbs.128084 (PMC13181855; doi:10.7150/ijbs.128084)
Supplement: Supplementary file 1 — Supplementary figures and tables. [file ijbsv22p4441s1.pdf]

List of Supplementary information for ‘**Loss of BATF3 impairs adipose–liver homeostasis and accelerates the transition from steatosis to fibrosis in high-fat diet–fed mice**’

Supplementary Tables……………………………………………………………..…p2-4

Supplementary Figure Legends and Figures…………………………………………p5-16

Supplementary Table 1, List of gene primers.

Genes	Forward	Reverse
**Lipid metabolism**		
**Pparα**	CTGTCGGGATGTCACACAAT	CAGGTCGTGTTCACAGGTAAG
**Pparγ**	CTGGCCTCCCTGATGAATAAA	AGGCTCCATAAAGTCACCAAA
**Srebpf1**	CGACTACATCCGCTTCTTACAG	AAGCTGACACCAGGTCTTTC
**Dgat1**	TCCGTCCAGGGTAGTG	TGAACAAAGAATCTTGCAGACGA
**Dgat2**	GCGCTACTTCCGAGACTACTT	GGGCCTTATGCCAGGAAACT
**Scd1**	TAGCACCTTCTTGCGATACAC	CTCCCGGGATTGAATGTTCTT
**Acc**	CCCAGCAGAATAAAGCTACTTTGG	TCCTTTTGTGCAACTAGGAACGT
**Fasn**	CGTGTGACCGCCATCTATATC	GATACCACCAGAGACCGTTATG
**Fabp5**	GCCAAGCCAGACTGTATCATTA	CTCCCAGGTTACAAGAGAACAC
**Cd36**	TGCTCTCCCTTGATTCTGCTGC	TTTGCTGCTGTTCTTTGCCACG
**Fatp2**	CTGGACAAAGTAGACGGAGTG	CCTGTGGTTCCCGAAGTATAAA
**Fatp5**	CCCAAAGCCAGCCATCTTA	GGACGTCATAGACCACATCATC
**Acsl1**	AGGACTCGGCATGTGACAAA	ACACCGCAGCAGAATCAGAA
**Acsl3**	CCTGTCAGTTCCAAACCGGA	CCAAAGTCAAGGGCTCGGAT
**Acsl4**	AGAGTCCAAAGCGAGGGAGA	TCTCTCCAGTTCCCAAACGC
**Acsl5**	TTAAACTTGGCGGGGTGAGA	CTGTGTAGCTCCTTTCGCCA
**Acox1**	TCCAGACTTCCAACATGAGGA	CTGGGCGTAGGTGCCAATTA
**Nox2**	ATGAGTTCCACACCTTCCTTC	GGCTTGAGACAACCTGGTATTA
**Nox4**	CCAGAATGAGGATCCCAGAAAG	GGTAGAAGCTGTAACCATGAGG
**Glucose metabolism**		
**Gck**	GAATCTTCTGTTCCACGGAGA	CAGAGTGCTCAGGATGTTAAGG
**Hk1**	GGCCAAGGAAAGCCTCTTAT	ACGCCTTCCTTATCCGTTTC
**Hk2**	GCTGGAGGTTAAGAGAAGGATG	TGGAGTGGCACACACATAAG
**Fbp1**	ACCCTGCCATCAATGAGTATC	ATACCAGAGTGCGGTGAATATC
**G6pc**	GTGGCAGTGGTCGGAGACT	ACGGGCGTTGTCCAAAC
**Pck1**	TTTGTAGGAGCAGCCATGAG	CCGAAGTTGTAGCCGAAGAA
**Slc2a2**	CACATCCTACTTGGCCTATCTG	CTTTGCCCTGACTTCCTCTT
**Hepatokines**		
**Activin-E**	GGAGGGATACCAGCTGAATTA C	CTACTACCATGTCTGGCACATC
**Angptl3**	GGCAGTCACGAAACCAACTA	TCCAGTAGATCCCTCTTCTTCTC
**Angptl4**	TTCCCTGCCCTTCTCTACTT	GGTAGCCTGCAGAGGATAGTA
**Angptl6**	TGGCAGAGTGGAGTGTATGA	CAAGGAGAAGCTGTCGTAGTG
**Angptl8**	GTCCAGAGCCAGCTCAAT ATG	GAAGGTGTAAAGCGTCCTCTTC
**Fetuin-A**	TGGCAAACTGTTCTGTGAGGC	CCGGGAAATCTCCACCAGTT
**Follistatin**	GTGACAATGCCACATACGCC	CTTCCGAGATGGAGTTGCAAGA
**Gdf15**	AACTGAGGTTCCTGCTGTTC	TCTCACCTCTGGACTGAGTATC
**Igf1**	GTCGTCTTCACACCTCTTCTAC	CTCATCCACAATGCCTGTCT

**Fgf21**	GCATACCCCATCCCTGACTC	AGGTGGGCTTCAGTGTCTTG
**Lect2**	GTGCCAGCAAATCTTCCAAC	CAGACAGTCGAATGCCATCA
**Sepp1**	TGGAAGACCTGCGCATAA A	GGGAATGTGAGGAAGGAGTAAG
**Smoc1**	TCGAGGTCGATGCAAAGATG	CGGTAACTGGACCTGAACATAC
**RPS17**	GGAGATCGCCATTATCCCCA	ATCTCCTTGGTGTCGGGATC
**Human ANGPTL8**	GTCTCTATGGCCGCACAATA	GCAGAATATCCTCCTCCATCTG
**Human 18S**	GTAACCCGTTGAACCCCATT	CCATCCAATCGGTAGTAGCG

Supplementary Table 2, List of protein antibodies.

Antibodies	Source	Cat. #
**PPARγ**	Proteintech	16643-1-AP
**ACOX1**	Proteintech	10957-1-AP
**NOX2**	Proteintech	19013-1-AP
**DGAT1**	Proteintech	82945-1-RR
**APOB**	Proteintech	20578-1-AP
**CD36**	Santa Cruz Biotechnology	SC-9154
**ATF4**	Cell Signaling Technology	11815S
**CHOP**	Cell Signaling Technology	5554S
**p-AKT(T308)**	Cell Signaling Technology	13038S
**ATGL**	Cell Signaling Technology	2138S
**ACSL1**	Cell Signaling Technology	4047S
**4-HNE**	Alpha Diagnostic	HNE11-S
**Collagen I**	Millipore	AB765p
**MPO**	LSBio	LS-B6699-250
**ANGPTL8**	R&D Systems	MAB8548
**rhANGPTL8**	R&D Systems	10159-AN
**rhTNF-alpha**	R&D Systems	10291-TA
**Flow cytometry**		
**CD16/32**	Biolegend	101320
**CD45**	Biolegend	103108
**CD11b**	Biolegend	101207
**Ly6C**	Biolegend	128026
**Ly6G**	Biolegend	127618
**7aad**	Biolegend	420404

Supplementary Fig. 1

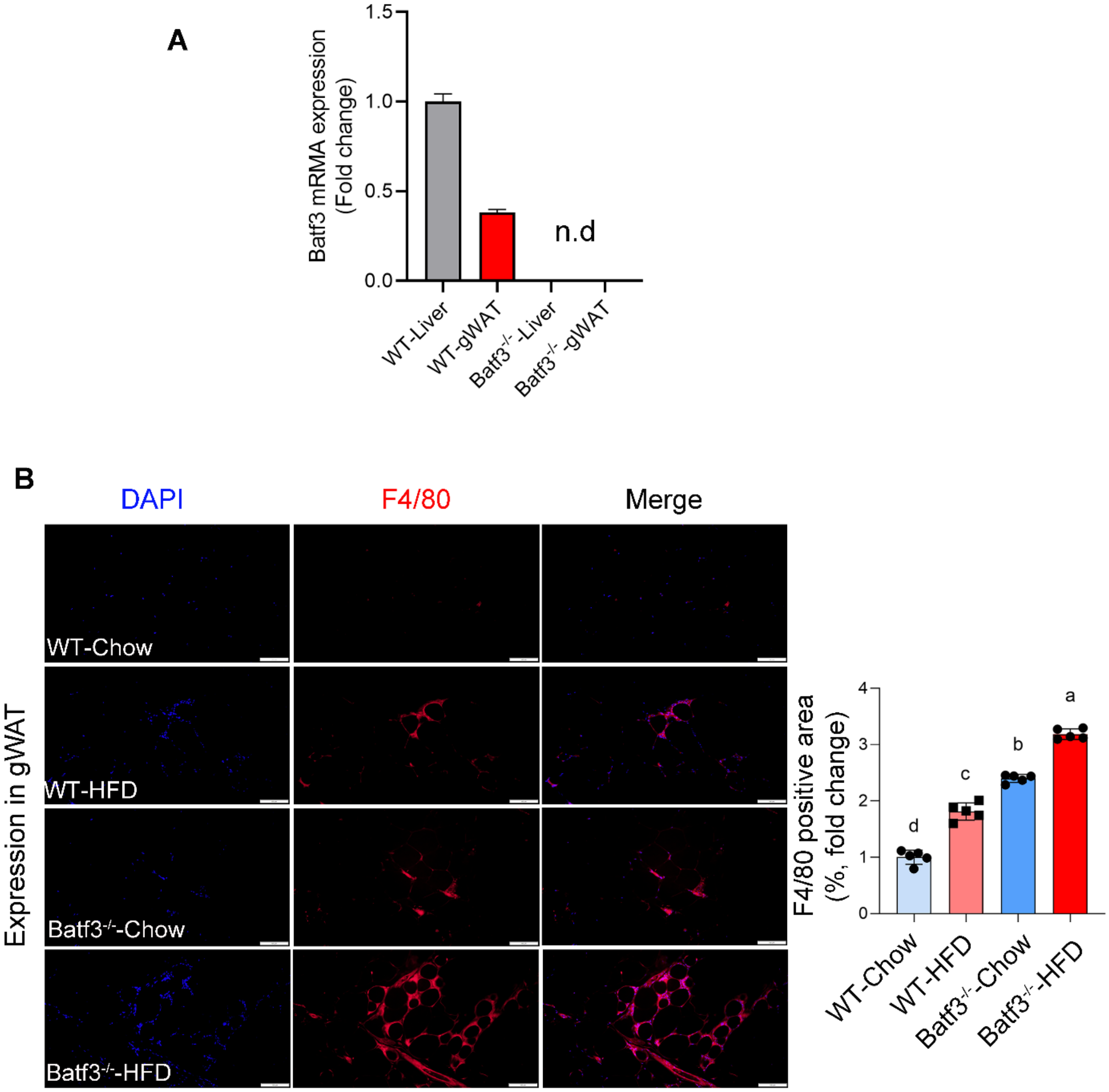

Fig. S1: Validation of BATF3 deficiency and immune phenotypes in adipose tissue. (A) Genotyping of Batf3 knockout (Batf3^{-}/$^{-}$) mice. Relative mRNA expression of batf3 was presented. n.d: no detected. (**B)** IF staining of F4/80 in gonadal white adipose tissue (gWAT) showing enhanced macrophage activation in Batf3^{-}/$^{-}$ mice compared with WT controls under chow and HFD feeding. Scale bar, 100 μm. Data are presented as mean ± SD. Different letters indicate statistically significant differences between groups.

Supplementary Fig. 2

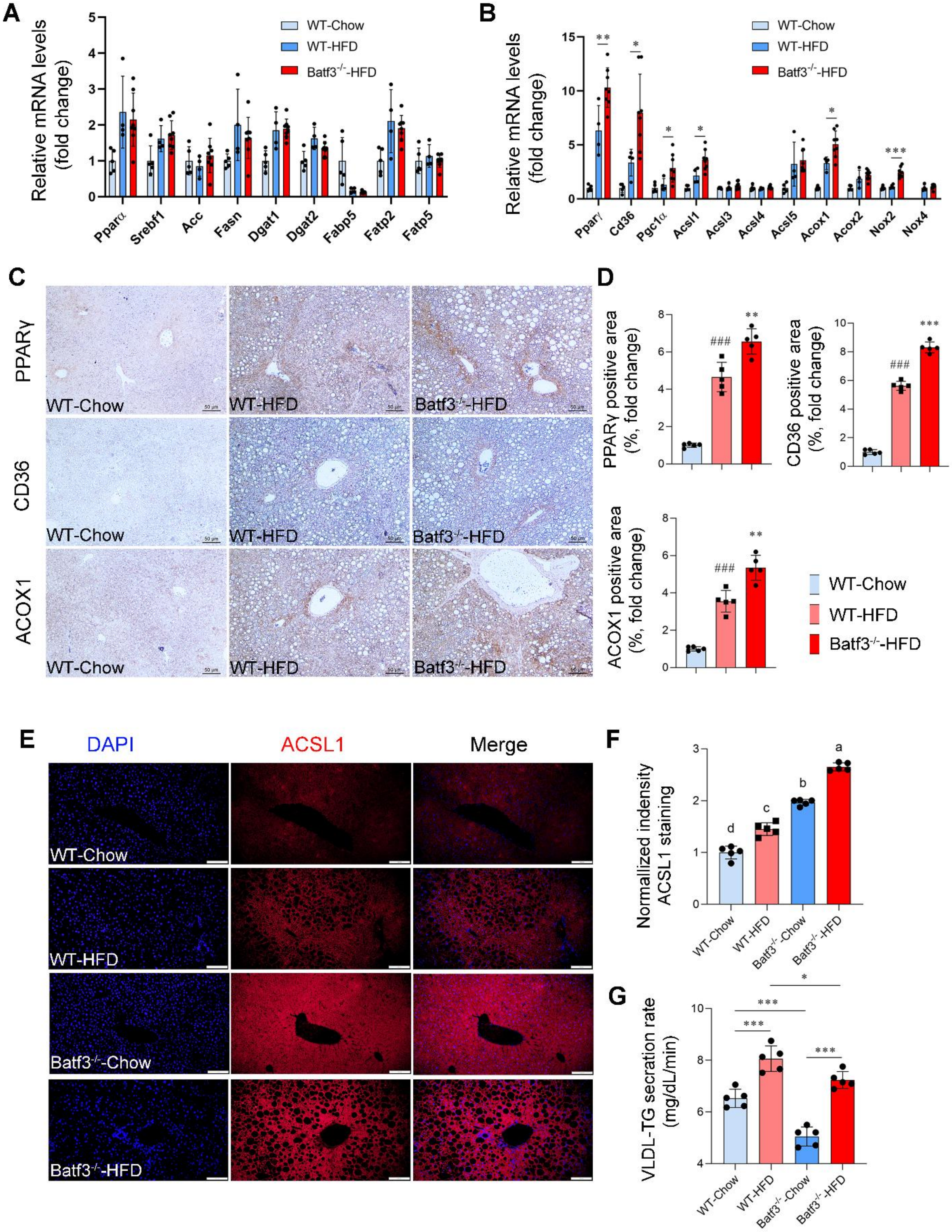

Fig. S2: BATF3 deficiency perturbs lipid metabolic gene expression and aggravates β-oxidation in the liver of HFD-fed mice. (A) qPCR analysis of hepatic expression of lipogenic genes (Pparα, Srebpf1, Fasn, Acc, Dgat1, Dgat2, Fabp5, Fatp2, Fatp5) in Batf3^{-}/$^{-}$ mice after HFD feeding. **(B)** qPCR analysis of PPARγ and CD36, key regulators of fatty acid uptake and storage, indicating enhanced lipid influx and retention. In parallel, the expression of β-oxidation–related genes, including Acsl1 and Acox1, was also elevated, suggesting a compensatory increase in fatty acid catabolism in response to lipid overload. **(C-D)** IHC staining and quantification of PPARγ, CD36, and ACOX1 in the livers of Batf3^{-}/$^{-}$ mice compared with WT controls. Scale bar, 500 µm. **(E-F)** IF staining and quantification of ACSL1 in the mouse liver. Scale bar, 100 µm. **(G)** VLDL-triglyceride secretion assay in WT and Batf3^{-}/$^{-}$ mice. Batf3^{-}/$^{-}$ mice displayed a marked reduction in circulating VLDL–TG accumulation compared with WT controls, indicating impaired hepatic triglyceride secretion. Mice were fed a high-fat diet for 16 weeks. Gene expression levels were quantified by qPCR and normalized to housekeeping genes using the ΔΔCt method. WT-Chow was used as the reference group and set to a value of 1. Data represent mean ± SD. *$P < 0.05$, **$P < 0.01$ vs. WT. Different letters indicate statistically significant differences between groups.

Supplementary Fig. 3

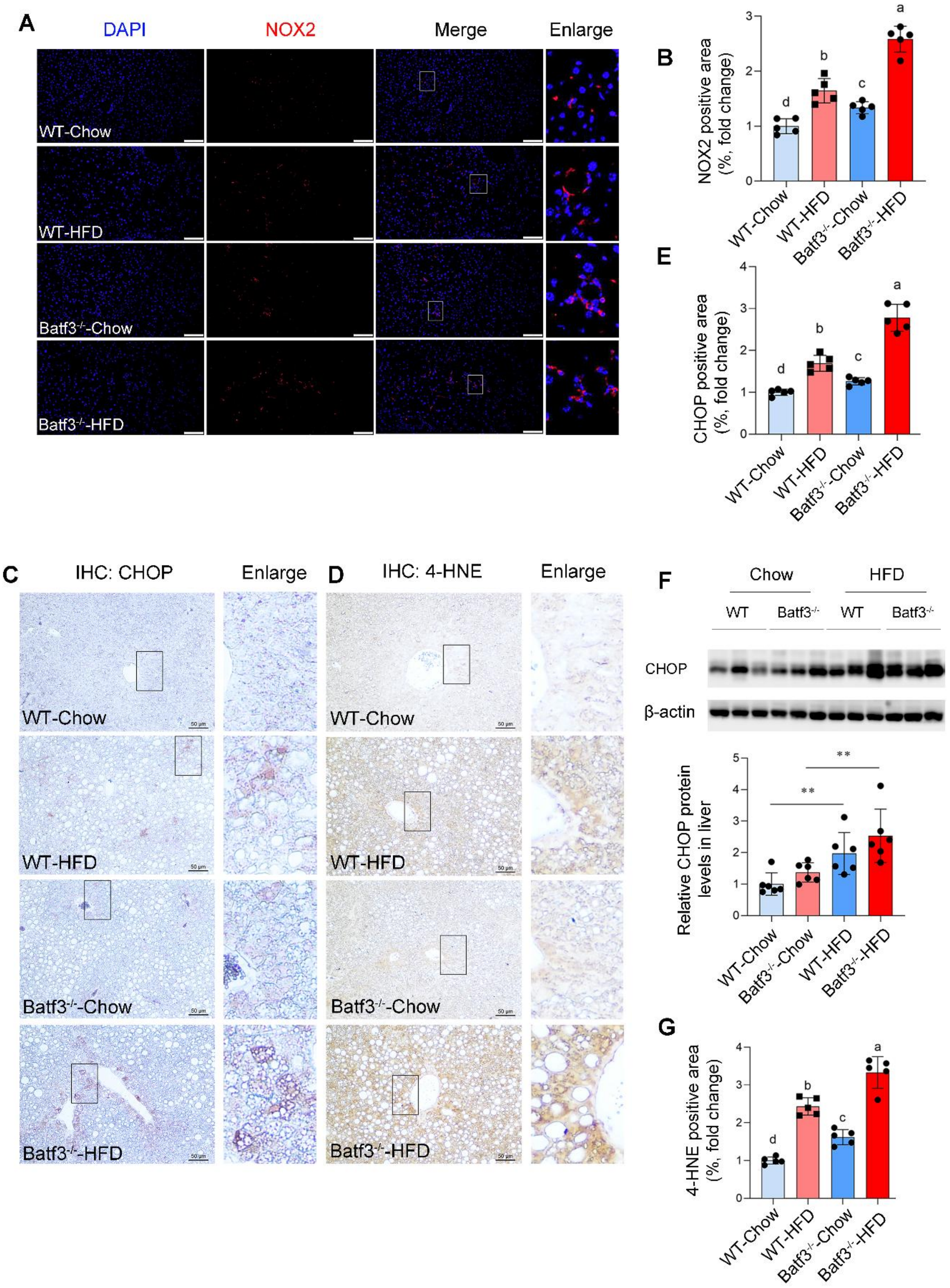

Fig. S3: BATF3 deficiency exacerbates inflammation and oxidative stress response in the liver of HFD-fed mice. (A-B) IF staining of NOX2 in liver tissue showing enhanced NOX2 expression in Batf3^{-}/$^{-}$ mice compared with WT controls under chow or HFD feeding. Scale bar, 100 μm. (**C-D)** IHC showing increased CHOP and 4-HNE expression in Batf3^{-}/$^{-}$ mice compared with WT under chow or HFD feeding. Scale bar, 50 μm. **(E)** Quantification of CHOP-positive area in liver tissue. **(F)** Western blot analysis of CHOP expression in the liver tissue. **(G)** Quantification of 4-HNE-positive area in liver tissue. Data are presented as mean ± SD. Different letters indicate statistically significant differences between groups.

Supplementary Fig. 4

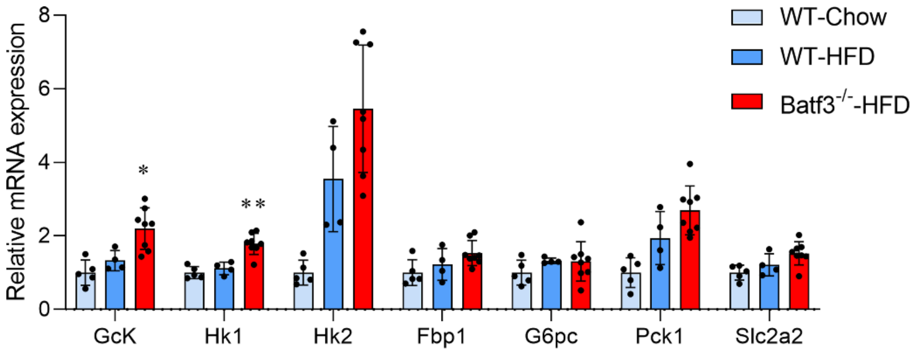

Fig. S4: Hepatic glucose metabolism gene expression in HFD-fed mice. qPCR analysis of gluconeogenic genes (G6pc, Pck1), glycolytic genes (Gck, Hk1, Hk2), and glucose transporter Slc2a2. Gene expression was quantified by qPCR and normalized to housekeeping genes using the ΔΔCt method. WT-Chow was used as the reference group and set to a value of 1. Data are shown as mean ± SD. **P* < 0.05, ***P* < 0.01 vs. WT-HFD.

Supplementary Fig. 5

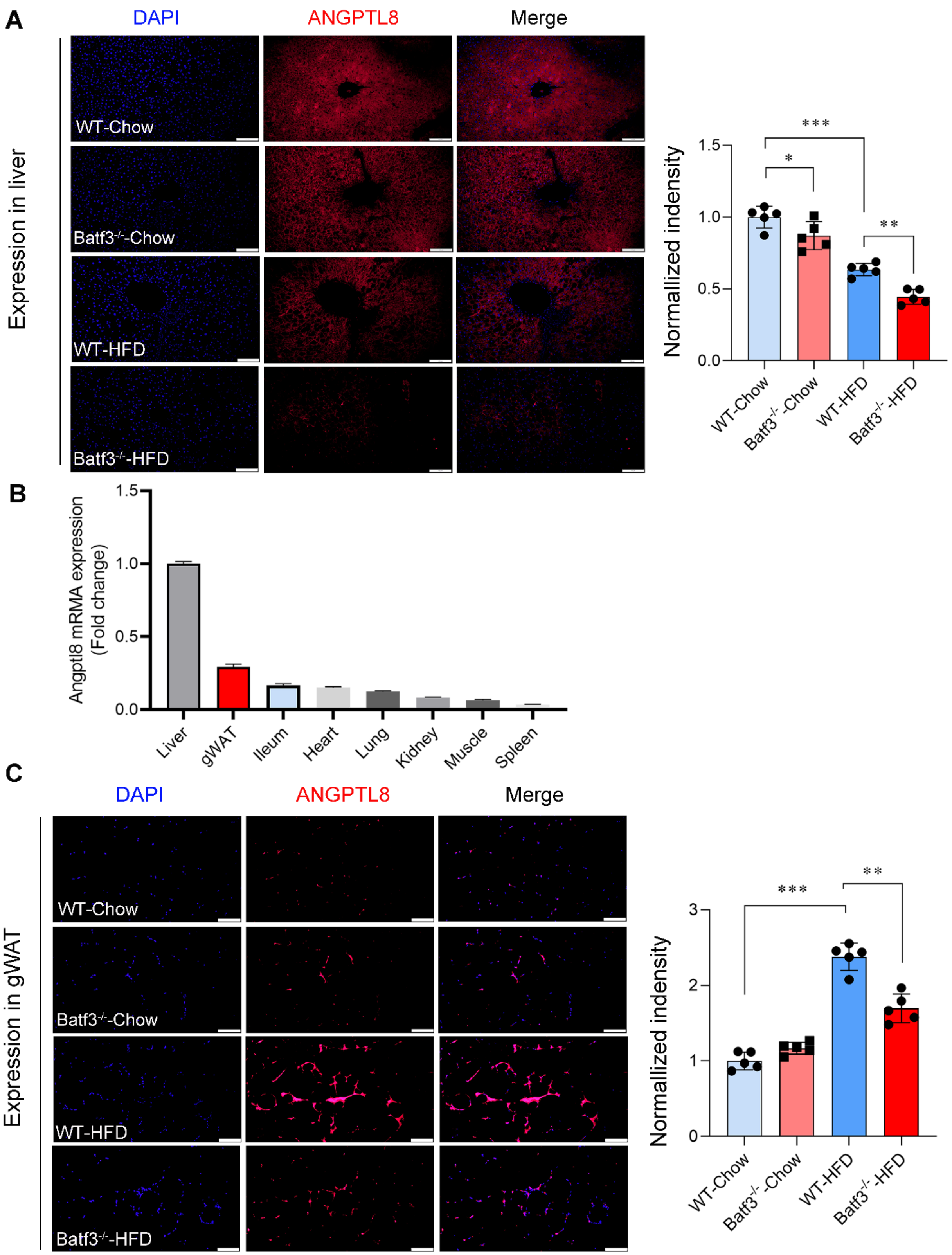

Fig. S5: ANGPTL8 expression in the liver and adipose tissue in WT and Batf3⁻/⁻ mice. (A) IF staining and quantification showed that ANGPTL8 expression was reduced in hepatocytes of Batf3⁻/⁻ mice. **(B)** qPCR analysis of ANGPTL8 mRNA expression across multiple tissues in normal adult male mice, with liver expression set as control. Data show higher enrichment in the liver and adipose tissue compared with other organs. **(C)** IF staining and quantification shows that ANGPTL8 expression was reduced in the gWAT of Batf3⁻/⁻ mice. Scale bar, 100 μm. Data represent mean ± SD. *$P < 0.05$, **$P < 0.01$, ***$P < 0.001$ vs. WT.

Supplementary Fig. 6

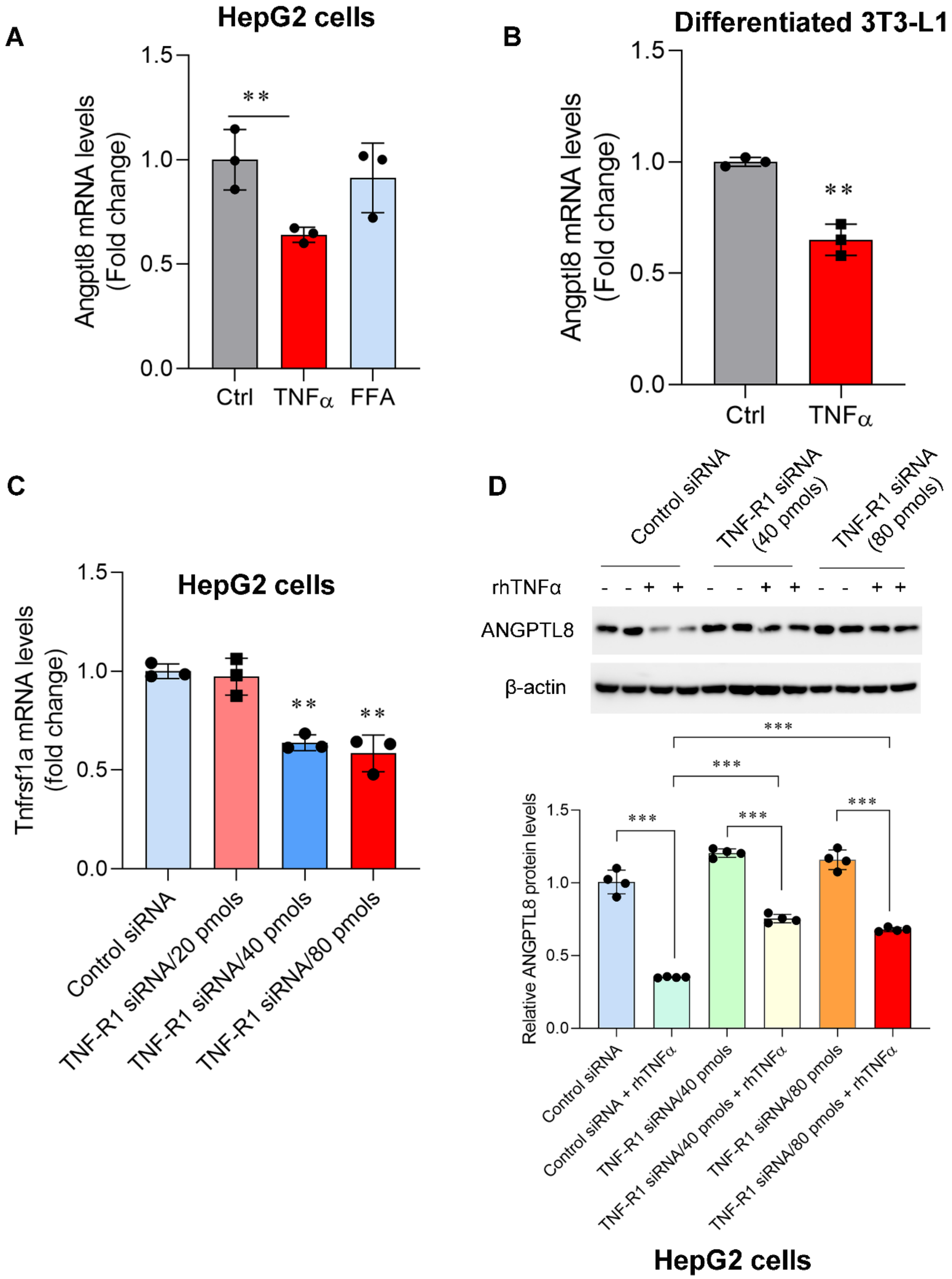

Fig. S6: Inflammatory signaling suppresses ANGPTL8 expression in the hepatocytes and adipocytes. (A) qPCR analysis of ANGPTL8 mRNA levels in HepG2 hepatocytes treated with free fatty acids (FFA) or TNFα. ANGPTL8 expression remained unchanged following FFA stimulation but was markedly reduced after TNFα treatment. **(B)** qPCR analysis of ANGPTL8 expression in differentiated 3T3-L1 adipocytes following TNFα stimulation, showing significant downregulation of ANGPTL8. **(C)** Efficiency of TNF receptor 1 (TNF-R1) knockdown in HepG2 cells transfected with siRNA targeting TNFRSF1A, confirmed by qPCR. **(D)** HepG2 cells were transfected with control siRNA or siTNFRSF1A and subsequently treated with TNFα. ANGPTL8 expression was analyzed by Western blot, n = 4/group. TNF-R1 knockdown significantly attenuated TNFα-induced downregulation of ANGPTL8, indicating that TNFα regulates ANGPTL8 through TNF-R1 signaling. Data represent mean ± SD. ***P* < 0.01, ****P* < 0.001 vs. control.

Supplementary Fig. 7

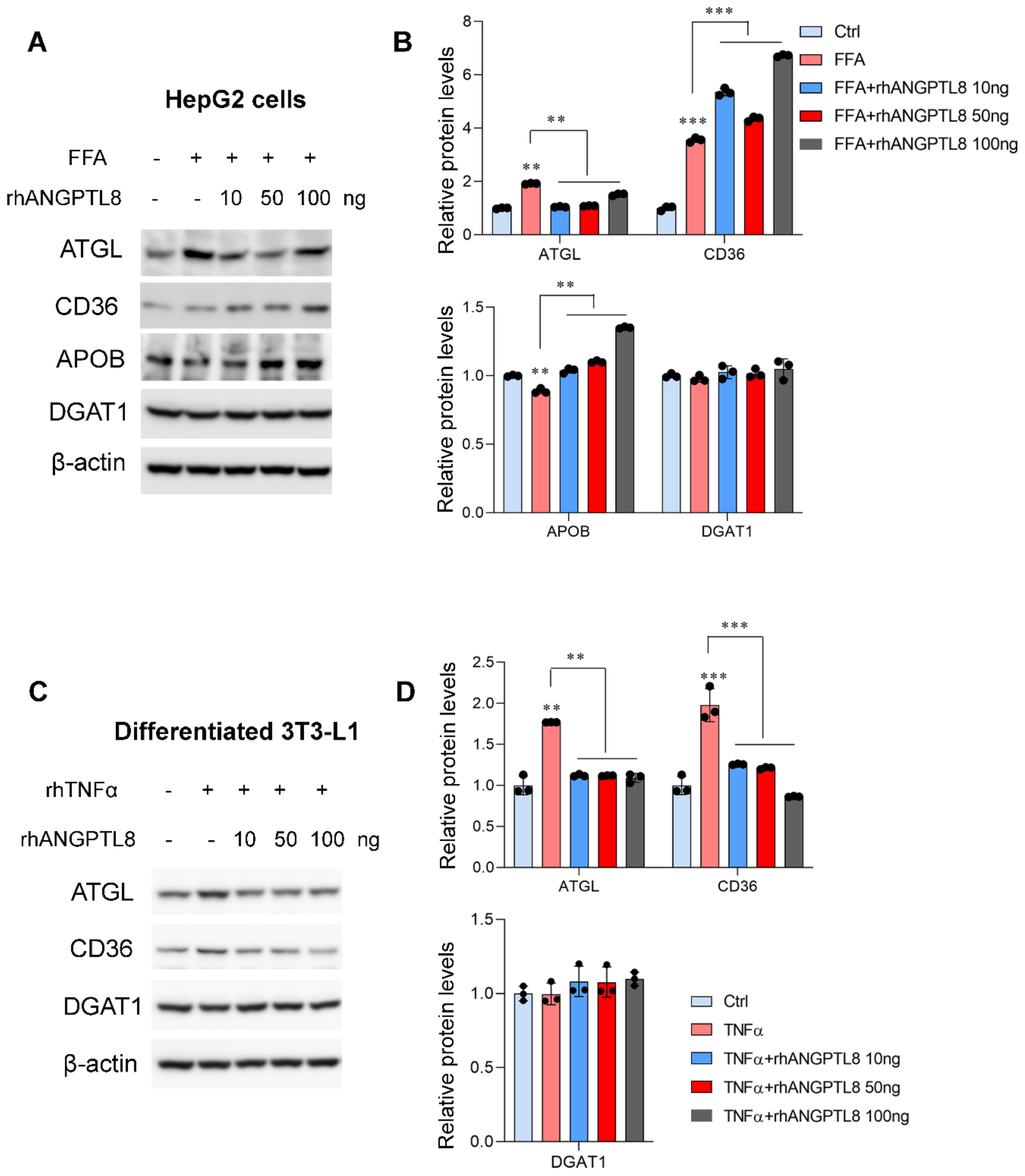

Figure S7. ANGPTL8 modulates triglyceride mobilization and export–related proteins in hepatocytes and adipocytes. (A-B) Representative Western blot analysis of ATGL, ApoB, and DGAT1 proteins in HepG2 cells treated with FFA in the presence or absence of recombinant ANGPTL8. **(C-D)** Representative Western blot analysis of ATGL and DGAT1 proteins in differentiated 3T3-L1 adipocytes treated with TNFα in the presence or absence of recombinant ANGPTL8. n =3/group. Data represent mean ± SD. **$P < 0.01$ vs. control.
